# A network comprising short and long noncoding RNAs and RNA helicase controls mouse retina architecture

**DOI:** 10.1038/ncomms8305

**Published:** 2015-06-04

**Authors:** Jacek Krol, Ilona Krol, Claudia Patricia Patino Alvarez, Michele Fiscella, Andreas Hierlemann, Botond Roska, Witold Filipowicz

**Affiliations:** 1 Neural Circuit Laboratories, Friedrich Miescher Institute for Biomedical Research, 4058 Basel, Switzerland; 2 Bio Engineering Laboratory, ETH Zurich, 4058 Basel, Switzerland; 3 Faculty of Medicine, University of Basel, 4056 Basel, Switzerland; 4 Biozentrum, University of Basel, 4056 Basel, Switzerland

## Abstract

Brain regions, such as the cortex and retina, are composed of layers of uniform thickness. The molecular mechanism that controls this uniformity is not well understood. Here we show that during mouse postnatal development the timed expression of Rncr4, a retina-specific long noncoding RNA, regulates the similarly timed processing of pri-miR-183/96/182, which is repressed at an earlier developmental stage by RNA helicase Ddx3x. Shifting the timing of mature miR-183/96/182 accumulation or interfering with Ddx3x expression leads to the disorganization of retinal architecture, with the photoreceptor layer being most affected. We identify Crb1, a component of the adhesion belt between glial and photoreceptor cells, as a link between Rncr4-regulated miRNA metabolism and uniform retina layering. Our results suggest that the precise timing of glia–neuron interaction controlled by noncoding RNAs and Ddx3x is important for the even distribution of cells across layers.

## Abstract (web summary)

The mammalian retina is a modular brain region, in which cell layers are of uniform thickness but the molecular mechanism controlling this process is not well understood. Here the authors identify a regulatory network consisting of the long noncoding RNA Rncr4, RNA helicase Ddx3x and miR-183/96/182 that controls the even distribution of cells across layers.

## Graphical abstract

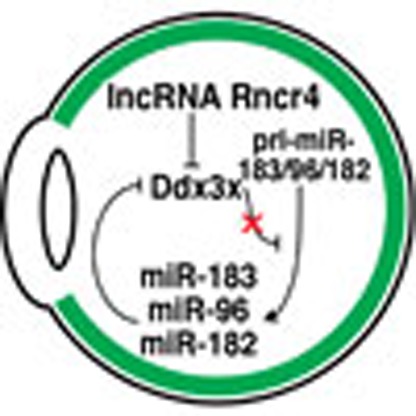

Many regions of the brain, such as the cortex, cerebellum and retina, are built up of repeated circuit modules. These modules have similar numbers of neurons, a feature that renders brain regions macroscopically uniform in appearance. One of the most studied modular regions of the mammalian brain is the retina, in which circuits are organized as vertical topographic domains. The cell bodies of the different cell types are also highly clustered, giving rise to so-called ‘nuclear' layers. The first such layer is the photoreceptor layer comprising cell bodies of rod and cone photoreceptors, the second, so-called inner nuclear, layer consists of cell bodies of bipolar, amacrine, horizontal and Müller cells, while the third layer, called ganglion cell layer, consists of ganglion and amacrine cell bodies. The thickness of each layer in most species is remarkably uniform, suggesting that the allocation of cells to each vertical domain is tightly controlled. However, the molecular mechanism controlling this process is not well understood.

At birth, mouse retina consists of a thick neuroblastic layer with immature rods and cones in the outer part. The photoreceptor layer becomes separated from the inner nuclear layer by P10. At the time the eyes open at P13–P14, cell proliferation and programmed cell death are complete and the photoreceptor layer has reached its final, highly uniform thickness^1^,^2^. Several genes have been shown to be necessary for uniform layer formation. Loss-of-function of the Crumbs-homologue protein Crb1 or atypical protein kinase C disturbs photoreceptor morphogenesis and leads to photoreceptor layer folding and disintegration^3^,^4^,^5^,^6^. Loss-of-function of the orphan nuclear receptor Nr2e3 or the basic motif-leucine zipper transcription factor Nrl leads to photoreceptor layer perturbation manifested by folds, whorls and pseudorosettes^7^,^8^.

Different classes of non-protein-coding RNAs, including long noncoding RNAs (lncRNAs; >200 nucleotides) or microRNAs (miRNAs; ∼21-nucleotide long), are dynamically expressed in eukaryotes in a developmental-, tissue- or cell type-specific manner and control diverse biological processes. LncRNAs have no defined common mode of action and may regulate gene expression and protein synthesis via chromatin remodelling and control of transcription, as well as post-transcriptionally at the level of mRNA editing, splicing, translation or degradation^9^. Several specific highly expressed lncRNAs are involved in retina development, but their mechanisms of action are largely unknown^10^,^11^. Knockdown or aberrantly timed expression of mouse Miat (also known as Rncr2—retinal noncoding RNA 2), Six3os1, Tug1 or Vax2os lncRNAs results in improper differentiation of photoreceptor progenitor cells and defects in cell type specification^12^,^13^,^14^,^15^.

MiRNAs control gene expression post-transcriptionally by base pairing to target mRNAs, causing inhibition of their translation as well as deadenylation and destabilization^16^. During biogenesis, the primary miRNA transcripts, pri-miRNAs, are trimmed by the Drosha/Dgcr8 complex into pre-miRNAs, which are further processed by Dicer into mature miRNAs^17^. Both the biogenesis and activity of miRNAs are the subjects of sophisticated control involving numerous protein–protein and protein–RNA interactions^18^. Recently, lncRNAs have also been shown to have an influence on miRNAs' activity^19^,^20^,^21^ and maturation^22^. The levels of one-third of retinal miRNAs change significantly during development, suggesting their potential involvement in the modulation of tightly controlled retinal maturation^23^,^24^,^25^. Depletion of all or particular miRNAs in developing mouse retina leads to aberrant photoreceptor layer architecture^26^, improper cell specification^27^, progressive photoreceptor degeneration^26^,^28^,^29^, functional defects^28^ or cell death^30^. A sensory-neuron-enriched miRNA cluster composed of three miRNAs (miR-183, miR-96 and miR-182; collectively referred to as miR-183/96/182) is predominantly expressed in mature photoreceptors^23^,^24^,^29^,^31^ to an extent modulated by different light regimes controlling processes of light/dark adaptation^32^. MiR-182 and miR-183 are also important in the maintenance of cone outer segments and cone function in adult mice^33^.

Here we investigate the relationship between the expression of long and short noncoding RNAs in photoreceptors during postnatal development. We find that the accumulation of mature miR-183/96/182 is delayed compared with pri-miR-183/96/182. We identify lncRNA Rncr4 (retinal noncoding RNA 4), expressed in maturing photoreceptors in the opposite direction to pri-miR-183/96/182, as a factor stimulating pri-miR-183/96/182 processing. Rncr4 modulates the activity of the DEAD-box RNA helicase/ATPase Ddx3x, which acts as an inhibitor of pri-miR-183/96/182 maturation in early postnatal photoreceptors. Disrupting the timing of miR-183/96/182 formation by enforcing early accumulation of mature miR-183/96/182 leads to profound irregularities in the thickness of the photoreceptor and inner nuclear layers. We find Crb1, a protein involved in the formation and integrity of tight junctions between retinal Müller glia and photoreceptors, to be regulated by miR-183/96/182. On the basis of the similarity of the effects on the organization of the photoreceptor layer of Crb1 knockout and forced early accumulation of miR-183/96/182 (refs 3, 6, 34), we propose that Rncr4-regulated timing of miR-183/96/182 metabolism controls the timed interaction of Müller glia and photoreceptor cells, and that this interaction ensures the even cell distribution in photoreceptor and inner nuclear layers.

## Results

### Similar patterns of Rncr4 and miR-183/96/182 expression

**Figure 1:**
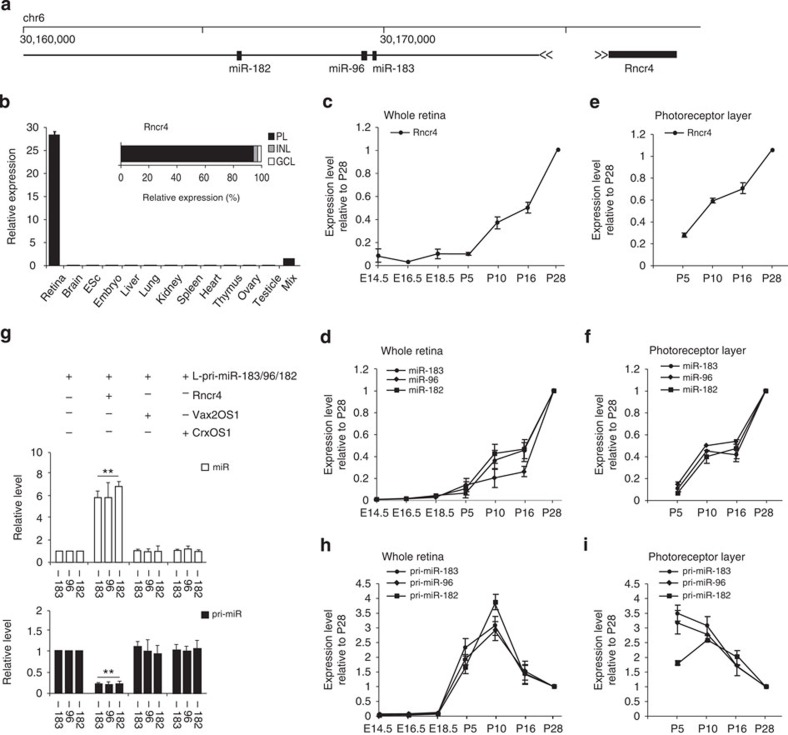
LncRNA Rncr4 accumulates parallel to miR-183/96/182 and stimulates pri-miR-183/96/182 processing. (**a**) Schematic representation of the mouse miR-183/96/182 gene locus with Rncr4 expressed as an opposite-strand transcript relative to pri-miR-183/96/182. (**b**) RT–qPCR analysis of Rncr4 shows strongly enriched expression in the retina when compared with other tissues; *n*=5. RT–qPCR analysis of Rncr4 levels in laser-dissected retinal cell layers reveals dominant expression of Rncr4 in photoreceptors. PL, photoreceptor layer; INL, inner nuclear layer; GCL, ganglion cell layer. (**c–f,h,i**) RT–qPCR analysis of Rncr4 and individual pri-miRNAs and mature miRNAs in developing retina or laser-dissected photoreceptors indicates inhibition of pri-miR-183/96/182 processing in early postnatal stages and progressive accumulation mature miR-183/96/182 paralleling expression of Rncr4 from P10 onwards. RNA levels at P28 are set to 1; *n*=10 retinas per group. (**g**) RT–qPCR analysis of mature miRNAs (white bars) and pri-miRNAs (black bars) in HEK293T cells expressing L-pri-miR-183/96/182 together with Rncr4, Vax2OS1 or CrxOS1 lncRNAs; *n*=5. Values in the absence of any co-expressed lncRNA are set to 1. **P<0.005; Mann–Whitney *U*-test. (**b–i**) Normalized values are mean±s.e.m. Primers specific for individual pri-miRNAs were used for pri-miR-183/96/182 analysis.

The *pri-miR-183/96/182* gene is an intergenic unit located on mouse chromosome 6 between the two protein-coding genes *Ube2H* and *Nrf1*. Inspection of the *pri-miR-183/96/182* locus revealed that lncRNA Rncr4 is transcribed in the opposite direction from a region upstream of the *pri-miR-183/96/182* gene (Fig. 1a). Reverse transcription quantitative–PCR (RT–qPCR) analysis of total RNA from different mouse tissues revealed highly enriched Rncr4 expression in the retina, similar to that of pri-miR-183/96/182 and mature miR-183/96/182 (Fig. 1b and Supplementary Fig. 1a,b). Moreover, as is the case of miR-183/96/182 (ref. 32), Rncr4 was predominantly expressed in photoreceptors compared with other retinal cell layers (Fig. 1b). Cell fractionation experiments performed with whole retina or the photoreceptor layer revealed the presence of Rncr4 in RNA isolated from both nuclear and cytoplasmic fractions (Supplementary Fig. 1c). Northern and 5′RACE (rapid amplification of cloned/cDNA ends) analyses identified Rncr4 as an ∼1.9-kb transcript (in contrast to the 1.4-kb GeneBank-annotated sequence, NR_038124; refs 35, 36) and mapped a proximal transcription start site ∼500 bp upstream of the annotated Rncr4 5′-end (Supplementary Fig. 1d,e).

RT–qPCR analysis of Rncr4 expression in retinas at embryonic (E) days 14.5, 16.5 and 18.5 and postnatal (P) days 5, 10, 16 and 28 revealed a pattern very similar to the accumulation of miR-183/96/182. Rncr4 and mature miRNAs were present at very low levels at embryonic and P5 stages and both increased gradually through P10–P28 (Fig. 1c,d). Since miR-183/96/182 is predominantly expressed in photoreceptors^29^,^31^, we used laser-capture microscopy together with RT–qPCR to determine the time course of Rncr4 and miR-183/96/182 accumulation in isolated photoreceptor layers (Fig. 1e,f). This analysis revealed developmental kinetics of accumulation similar to those obtained with whole retinas. We conclude that Rncr4 accumulates in retinal photoreceptors with developmental kinetics similar to those of sensory-neuron-enriched miR-183/96/182.

### Rncr4 stimulates processing of pri-miR-183/96/182

The similar accumulation profiles of Rncr4 and miR-183/96/182 may arise in at least two different ways. The accumulation of one could control accumulation of the other. Alternatively, the accumulation of both could be regulated by the same factors, for example, transcription factors influencing the expression of both Rncr4 and pri-miR-183/96/182. We investigated the relationship between Rncr4 and miR-183/96/182 expression first using HEK293T cells, which do not endogenously express either miR-183/96/182 or lncRNA (corresponding to Rncr4) at appreciable levels. The cells were co-transfected with plasmids expressing the central 5.5-kb region of pri-miR-183/96/182 (referred to as long (L)-pri-miR-183/96/182) encoding all three miR-183/96/182 miRNAs and either Rncr4, or one of two further retinal lncRNAs, Vax2OS1 or CrxOS (ref. 10) as controls. Surprisingly, co-expression of Rncr4 resulted in strongly enhanced processing of L-pri-miR-183/96/182. Mature miRNAs increased sixfold, while the level of L-pri-miR-183/96/182 significantly decreased, as determined using three sets of primers specific for individual pri-miRNAs. Co-expression of Vax2OS1 or CrxOS1 RNA had no effect on L-pri-miR-183/96/182 processing (Fig. 1g).

The results with HEK293T suggested that Rncr4 has a stimulatory effect on the processing of pri-miR-183/96/182 rather than on the transcription of its gene. To explore the link between Rncr4 and miR-183/96/182 biogenesis in a more physiological setting, we analysed the expression of pri-miR-183/96/182 in the mouse retina during development and compared it with the accumulation of mature miR-183/96/182 and Rncr4. Notably, pri-miR-183/96/182 levels in isolated retinas or laser-captured photoreceptor layers were already close to or at their maximum at P5 (Fig. 1h,i), that is, at the stage when the accumulation of both mature miR-183/96/182 and Rncr4 was just beginning (Fig. 1c–f). While pri-miR-183/96/182 levels decreased two- to threefold between P5 and P28, miR-183/96/182 and Rncr4 increased during that time by ∼10-fold and 4- to 10-fold, respectively. To determine whether the observed delay in accumulation of mature miRNAs was specific to pri-miR-183/96/182, we measured the levels of pri-miRNAs and mature miRNAs for neuronal miR-128 and ubiquitously expressed miR-16 in developing retina. No delays in processing, similar to that seen for miR-183/96/182, were observed for miR-128 or miR-16 (Supplementary Fig. 1f).

Taken together, the results of these *in vitro* and *in vivo* experiments support a model in which Rncr4 specifically stimulates conversion of pri-miR-183/96/182 to mature miR-183/96/182 in developing photoreceptors. The questions of the factor responsible for the delayed processing of pri-miR-183/96/182 and the mechanism by which Rncr4 contributes to the accumulation of miR-183/96/182 in the developing retina were then considered.

### Ddx3x inhibits pri-miR-183/96/182 processing

**Figure 2:**
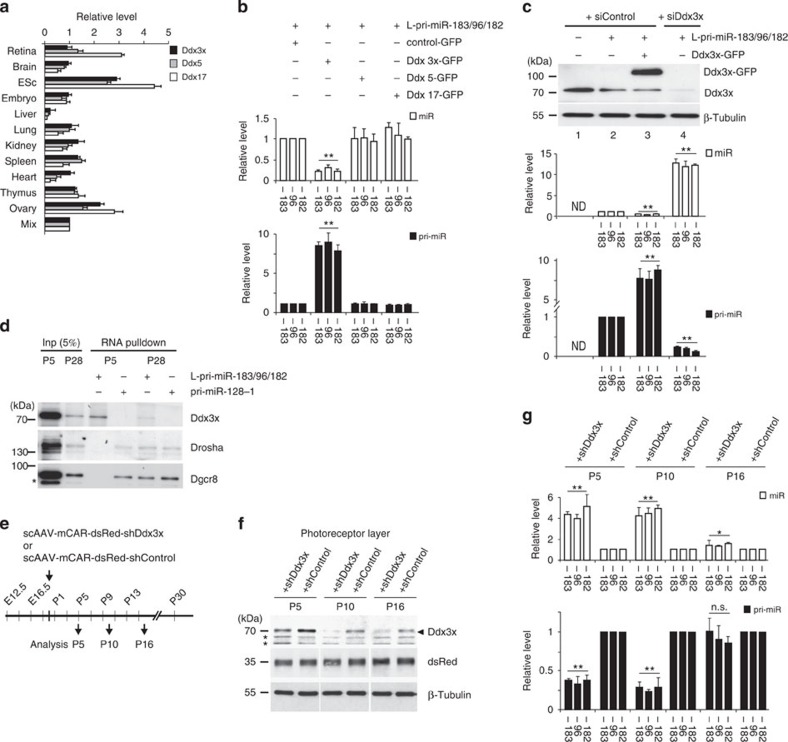
Ddx3x inhibits the pri-miR-183/96/182 processing. (**a**) RT–qPCR analysis of the Ddx3x, Ddx5 and Ddx17 mRNA levels in different mouse tissues; *n*=3. Values for mixture (Mix) of all RNA samples were set to 1. (**b**) RT–qPCR analysis of miR-183/96/182 and pri-miR-183/96/182 levels in HEK293T cells expressing L-pri-miR-183/96/182 and different helicases reveals repressive effect of Ddx3x, but not Ddx5 and Ddx17, on pri-miR-183/96/182 processing; *n*=3. (**c**) SiRNA knockdown of endogenous Ddx3x (see western blot analysis shown in the upper most panel) activates L-pri-miR-183/96/182 processing in HEK293T cells. Cells treated with control siRNA or a mixture of two Ddx3x-specific siRNAs (siRNA-I and siRNA-II) were co-transfected with plasmid expressing L-pri-miR-183/96/182. Lane 3 represents an additional control showing that overexpression of Ddx3x-GFP further represses L-pri-miR-183/96/182 processing; *n*=3. ND, non detectable. (**d**) RNA pull-down assay identifies proteins interacting with L-pri-miR-183/96/182 in extracts of P5 and P28 retinas. Pri-miR-128-1 served as a control. Asterisks indicate unspecific bands. (**e**) Schematic representation of experiments shown in **f**,**g**. Eyes were injected subretinally at P0 with scAAVs expressing, under photoreceptor-specific mCAR promoter, dsRed marker and either two Ddx3x-specific (Ddx3x-I and Ddx3x-II) or control shRNAs. Retinas were collected at P5, P10 and P16. (**f**) Western blot analysis of Ddx3x, dsRed and β-Tubulin levels in laser-dissected photoreceptors of P5, P10 and P16 retinas infected with scAAVs described in **e**. Asterisks indicate unspecific bands, which also provide an additional loading control. (**g**) RT–qPCR analysis of miR-183/96/182 and pri-miR-183/96/182 levels in photoreceptors of retinas infected with either scAAV-shDdx3x or scAAV-shControl. Values for shControl were set to 1. (**a–c,g**) Normalized values are means±s.e.m. (*n*=5). *P<0.05; **P<0.005; Mann–Whitney *U*-test.

We hypothesized that pri-miR-183/96/182 processing in the early postnatal retina is repressed by an inhibitor expressed in photoreceptors and that Rncr4 interferes with this inhibitor function in late postnatal and adult photoreceptors. The Drosha/Dgcr8-containing ‘microprocessor' complex includes many accessory proteins acting as either positive or negative regulators of miRNA biogenesis^18^. Among the microprocessor complex components are three members of the DEAD-box family of RNA-dependent ATPases/helicases: Ddx5, Ddx17 and Ddx3x (ref. 37); all these proteins are expressed in the retina (Fig. 2a).

We tested whether these DEAD-box proteins influence the biogenesis of miR-183/96/182. Plasmids expressing individual green fluorescent protein (GFP)-tagged proteins were transfected into HEK293T cells together with a vector expressing L-pri-miR-183/96/182. Expression of Ddx5 and Ddx17 had no significant effect on L-pri-miR-183/96/182 processing compared with the controls (Fig. 2b). In contrast, expression of Ddx3x had a pronounced inhibitory effect on the processing of L-pri-miR-183/96/182, resulting in a 10-fold increase in pri-miR-183/96/182 and a concomitant 5-fold decrease in mature miR-183/96/182 levels compared with controls. Moreover, short interfering RNA (siRNA)-mediated depletion of endogenous Ddx3x in HEK293T cells resulted in a markedly higher efficiency of L-pri-miR-183/96/182 processing, as manifested by increased accumulation of mature miR-183/96/182 and a decrease in pri-miRNA levels compared with controls (Fig. 2c).

To determine whether ATPase/helicase activity of Ddx3x is required for inhibition of L-pri-miR-183/96/182 processing, we knocked down endogenously expressed Ddx3x in HEK293T cells by siRNAs and expressed siRNA-resistant (siRes) Ddx3x mutants bearing single amino-acid substitutions within the GKT (motif I; MutK230E) and SAT (motif III; MutS382L) motifs. These mutations are known to impair ATPase and helicase functions of DEAD-box proteins, respectively^38^,^39^. In HEK293T cells depleted of endogenous Ddx3x, neither of the overexpressed Ddx3x mutants repressed L-pri-miR-183/96/182 processing, while wild-type Ddx3x efficiently rescued the repression (Supplementary Fig. 2). We conclude that Ddx3x acts as a repressor of pri-miR-183/96/182 processing in transfected HEK293T cells and that this function requires ATPase/helicase activity of the protein.

We next tested whether endogenous Ddx3x present in retinal extracts interacts with L-pri-miR-183/96/182. Immobilized L-pri-miR-183/96/182 was incubated with cell lysates of retinas from P5 and P28 mice and the L-pri-miR-183/96/182-bound proteins were analysed using western blot analysis. The RNA pull-down experiment identified Ddx3x as strongly bound and Drosha and Dgcr8 as weakly bound by L-pri-miR-183/96/182 at P5 (Fig. 2d). In contrast, L-pri-miR-183/96/182 pulled down less Ddx3x but more Drosha and Dgcr8 at P28 than at P5. Pri-miR-128-1 as a control associated with Drosha and Dgcr8 equally well at P5 and P28 but did not pull down Ddx3x at either developmental time point (Fig. 2d).

The specific association of pri-miR-183/96/182 with Ddx3x but not Drosha/Dgcr8 in extracts of the P5 retina suggested, as in HEK293T cells, that Ddx3x also inhibits pri-miR-183/96/182 processing in the retina. To test this further, we injected retinas at P0 with a self-complementary adeno-associated virus (scAAV) expressing either two different Ddx3x-specific or control short hairpin RNAs (shRNAs), driven by the photoreceptor-specific mCAR promoter^40^ (Fig. 2e). Use of scAAV allowed efficient shRNA expression already 3 days after injection compared with ∼10 days for a regular AAV^41^. The photoreceptor-specific knockdown of Ddx3x resulted in a threefold decrease in pri-miRNA and fivefold increase in mature miR-183/96/182 levels in photoreceptors of P5 and P10 retinas compared with control shRNA. At P16, Ddx3x knockdown increased mature miR-183/96/182 levels in photoreceptors by 1.5-fold over the control (Fig. 2f,g). We conclude that Ddx3x acts as a repressor of pri-miR-183/96/182 processing in early postnatal photoreceptors.

### Rncr4 antagonizes effect of Ddx3x on pri-miR-183/96/182

**Figure 3:**
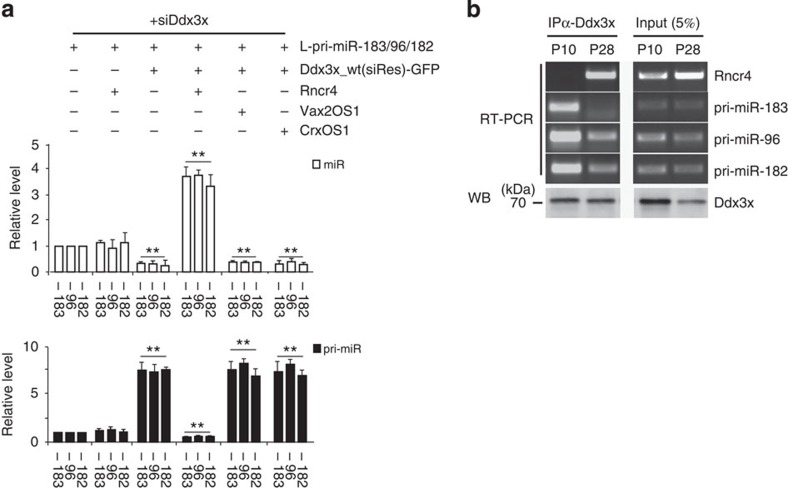
Rncr4 antagonizes inhibitory effect of Ddx3x on pri-miR-183/96/182 processing. (**a**) RT–qPCR analysis of miR-183/96/182 and pri-miR-183/96/182 levels in Ddx3x-depleted HEK293T cells. Cells were transfected with a mixture of two siRNAs (siDdx3x-I and -II) and plasmid expressing L-pri-miR-183/96/182. When indicated, transfections also included plasmids encoding siRNA-resistant Ddx3x, and Rncr4, Vax2OS1 or CrxOS1. *n*=5. (**b**) Anti-Ddx3x immunoprecipitation identifies Ddx3x-interacting RNAs in P10 and P28 retinal extracts. Immunoprecipitated RNAs were analysed with RT–PCR. Ddx3x distribution was analysed with western blot analysis.

Having established that Rncr4 acts as an activator and Ddx3x as an inhibitor of pri-miR-183/96/182 processing, we investigated the mechanisms involved, first by studying the relationship between Ddx3x and Rncr4 in HEK293T cells. As indicated above, in HEK293T cells depleted of endogenous Ddx3x, expression of siRes wild-type Ddx3x but not its ATPase/helicase mutants resulted in significant overaccumulation of co-expressed L-pri-miR-183/96/182 and a decrease in the level of mature miRNAs (see Supplementary Fig. 2). We now found that co-expression of wild-type Ddx3x and Rncr4 in HEK293T cells resulted in strongly enhanced processing of L-pri-miR-183/96/182, with a significant fourfold increase in mature miRNAs and a fivefold decrease in L-pri-miR-183/96/182 levels compared with the controls (Fig. 3a). The effect of Rncr4 was specific since two other retinal lncRNAs, Vax2OS1 and CrxOS1, had no effect on L-pri-miR-183/96/182 processing. We conclude that Rncr4 has the potential to antagonize the repressive effect of Ddx3x on pri-miR-183/96/182 processing.

To determine whether Rncr4 interacts with Ddx3x in the retina, we performed RNA immunoprecipitation assays using an anti-Ddx3x antibody and extracts from P10 and P28 retinas; P28 retinas contain high levels of Rncr4 but this RNA is already present in a substantial amount at P10 (Figs 3b and 1c,e). Notably, RT–PCR identified Rncr4 as an RNA associated with Ddx3x at P28 but much less so at P10 (Fig. 3b). Consistent with the results of previous RNA pull-down assays (see Fig. 2d), RT–PCR also revealed a markedly higher association of Ddx3x with pri-miR-183/96/182 in P10 than in P28 retinas (Fig. 3b).

Taken together, our data indicate that Rncr4 acts as an inhibitor of Ddx3x while Ddx3x represses pri-miR-183/96/182 processing. Hence, by antagonizing the repressive effect of Ddx3x, Rncr4 is an activator of pri-miR-183/96/182 processing in late postnatal photoreceptors (see Discussion).

### Ddx3x is a target of miR-183/96/182 in photoreceptors

Computational predictions identified Ddx3x mRNA as a potential target of miR-183, miR-96 and miR-182, bearing two conserved miRNA-binding sites in the 3′-untranslated repeat (UTR; Supplementary Fig. 3a), suggesting that miR-183/96/182 may act as a feedback signal controlling Ddx3x levels. We used reporters with a firefly luciferase (FL)-coding sequence fused to the Ddx3x 3′-UTR, containing either wild-type or mutant miRNA-binding sites, to test a potential inhibitory influence of miR-183/96/182 on Ddx3x mRNA function. Compared with the pFL-Ddx3x_mut_3′UTR reporter, FL activity of the pFL-Ddx3x_wt_3′UTR reporter was strongly and significantly reduced by the miR-183/96/182 mimics transfected into HEK293T cells either individually or in combination (Supplementary Fig. 3b).

We then asked whether miR-183/96/182 inhibits Ddx3x expression in photoreceptors *in vivo*. To this end, we first performed western blot analysis using extracts of laser-dissected photoreceptor layers from P5–P28 retinas. The Ddx3x protein level decreased gradually in photoreceptors between P5 and P28 (Supplementary Fig. 3c), consistent with a gradual accumulation of miR-183/96/182 (see Fig. 1f). We then tested whether inhibition of miR-183/96/182 activity *in vivo* increases Ddx3x expression by injecting retinas at P0 with AAV expressing a miR-183/96/182 triple sponge and enhanced GFP (EGFP) marker under the photoreceptor-specific mCAR promoter (Supplementary Fig. 3d). The triple sponge was previously shown to effectively upregulate the investigated miR-183/96/182 targets in mouse retina^32^. Western blot analysis of protein extracts from the laser-dissected photoreceptors of P28 retina revealed that infection with AAV-mCAR-EGFP-triple sponge resulted in a marked increase in Ddx3x protein level compared with retina infected with the AAV-mCAR-EGFP/control, indicating that Ddx3x is also regulated by miR-183/96/182 *in vivo* (Supplementary Fig. 3e). Interestingly, in P28 photoreceptors of the P0 sponge-infected retina, the increase in Ddx3x had no significant impact on the efficiency of pri-miR-183/96/182 processing, as indicated by measurements of pri- and mature miR-183/96/182 levels (Supplementary Fig. 3f). Possibly, in photoreceptors of the late postnatal or adult retina the Ddx3x regulatory function in processing of pri-miR-183/96/182 is suppressed by abundantly expressed Rncr4. Alternatively, the Ddx3x inhibitory effect on pri-miR-183/96/182 processing may involve a further cofactor present in early postnatal but not late postnatal or adult photoreceptors. Taken together, our findings suggest that Ddx3x and miR-183/96/182 form a reciprocal-negative regulatory loop that mutually influences their levels and activities in developing photoreceptors.

### Premature miRNA accumulation affects retina architecture

**Figure 4:**
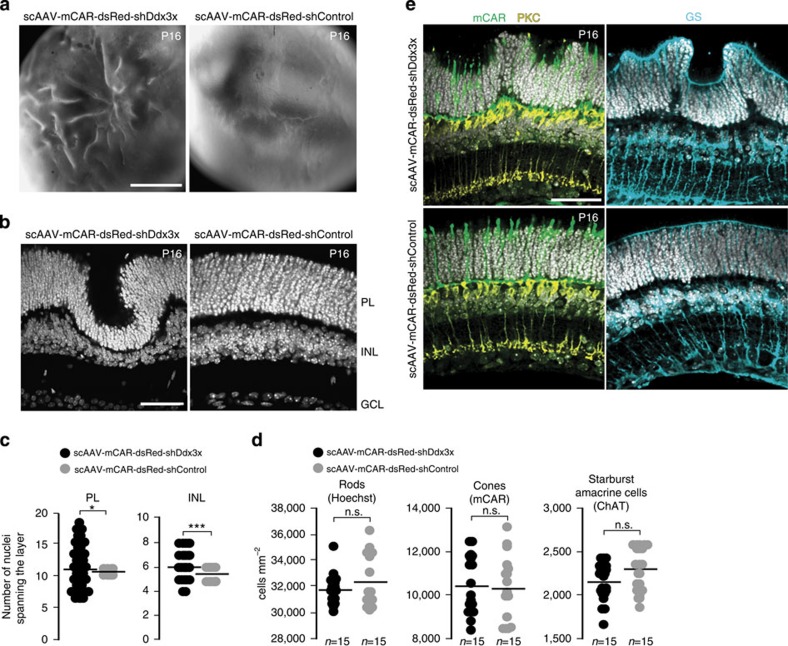
Ddx3x knockdown in early postnatal photoreceptors leads to an increased variation in photoreceptor layer thickness. (**a**) Bright field images of P16 retinas infected with scAAV-mCAR-dsRed-shDdx3x or -shControl viruses. (**b**) Representative image of Hoechst-stained (white) vertical section of P16 shDdx3x-treated retina shows wavy appearance when compared with ShControl. (**c**) Quantification of photoreceptor layer (PL) and inner nuclear layer (INL) thicknesses of P16 shDdx3x-treated or control retinas. *n*=50. *P<0.05; ***P<0.001; Mann–Whitney *U*-test. (**d**) Quantifications of rods, cones and starburst amacrine cells, stained for indicated markers, in P16 retinas infected with scAAV-mCAR-dsRed-shDdx3x or -shControl viruses. n.s. not significant; Mann–Whitney *U*-test. (**e**) Confocal images of vertical sections of P16 shDdx3x-treated or shControl retinas stained with antibodies against cone arrestin (mCAR), protein kinase C (PKC) and glutamine synthetase (GS) to label cones, ON bipolar cells and Müller cells, respectively. Hoechst-stained nuclei are in white. PL, photoreceptor layer; INL, inner nuclear layer; GCL, ganglion cell layer. Scale bars, (**a**) 500 μm; (**b,e**) 50 μm.

The first indication of a possible biological role for the delay in the formation of mature miR-183/96/182 in developing photoreceptors came from the examination of mice subjected to photoreceptor-specific knockdown of Ddx3x at P0. This resulted in a significant increase in mature miR-183/96/182 levels already at P5, that is, at a time when mature miR-183/96/182 are normally very low (see Fig. 2g). Analysis of P16 retinas from these mice revealed dramatic changes in retinal architecture, with the photoreceptor and inner nuclear layers displaying a wavy appearance with large protrusions and valleys (Fig. 4a,b).

**Figure 5 f5:**
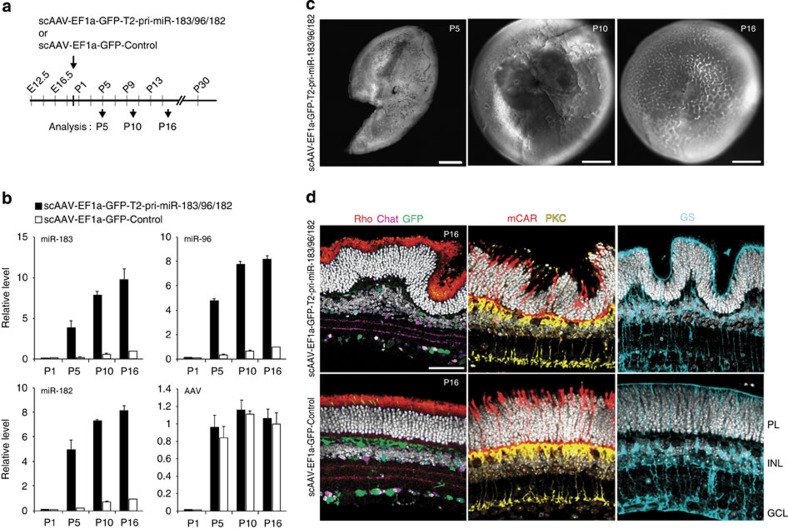
Premature miR-183/96/182 accumulation leads to changes in the retinal architecture. (**a**) Schematic representation of experiments. Eyes were injected subretinally at P0 with scAAVs expressing GFP and either T2-pri-miR-183/96/182 or control RNA. (**b**) RT–qPCR analysis of miR-183/96/182 and AAV levels in T2-pri-miR-183/96/182 and control retinas at P5, P10 and P16. Values, normalized to controls at P16 set as 1, are means±s.e.m. *n*=3. (**c**) Bright field images indicating onset and progression of the phenotype in T2-pri-miR-183/96/182 retinas. (**d**) Confocal images of vertical sections of P16 T2-pri-miR-183/96/182 or control retinas stained with antibodies against rhodopsin (Rho), choline acetyltransferase (Chat), mCAR, PKC and GS to label rods, starburst amacrine cells, cones, ON bipolar cells and Müller cells, respectively. Hoechst-stained nuclei are in white. PL, photoreceptor layer; INL, inner nuclear layer; GCL, ganglion cell layer. Scale bars, (**c**) 500 μm; (**d**) 50 μm.

To investigate the cellular architecture of the Ddx3x knockdown retina in more detail, we examined retinal sections using confocal microcopy. In wild-type mice, the number of cell nuclei stacked within individual retinal layers is known to be uniform. In the Ddx3x knockdown retinas, the number of photoreceptor nuclei spanning the photoreceptor layer at P16 ranged from 6 to 18 compared with 10–11 nuclei in control retinas (Fig. 4c). The width of the inner nuclear layer was also affected, with four to eight nuclei in the Ddx3x-knockdown retina compared with five to six nuclei in the control; however, on the basis of the observation that interplay between ncRNAs and Ddx3x occurs in photoreceptors, it is likely that the inner nuclear layer effect is a consequence of changes in organization of the photoreceptor layer. Architecture of inner plexiform and ganglion cell layers was not affected (Fig. 4b,e; see also Fig. 5d). Importantly, no significant changes were observed in the total numbers of different retinal cell types, in P16 Ddx3x-knockdown retinas compared with the control, including rods (counted nuclei stained with Hoechst), cones (stained for cone arrestin), starburst amacrine cells (choline acetyltransferase), activated by light increments ON bipolar cells (protein kinase C) and Müller cells (glutamine synthetase; Fig. 4d,e).

To test whether the increased variance in photoreceptor layer thickness in Ddx3x-knockdown retina can be explained by premature formation of miR-183/96/182, we precociously induced miR-183/96/182 accumulation. For this, we engineered a pri-miR-183/96/182 form that is immune to Ddx3x-mediated repression. Using HEK293T cells, we tested several variants of L-pri-miR-183/96/182 containing terminal and/or internal deletions and identified a truncated 0.5-kb-long version of L-pri-miR-183/96/182 termed T2-pri-miR-183/96/182 (Supplementary Fig. 4a), which was not subject to inhibition even in cells overexpressing Ddx3x (Supplementary Fig. 4b). We then injected retinas at P0 with scAAVs expressing T2-pri-miR-183/96/182 under control of either ubiquitous EF1a or photoreceptor-specific mCAR promoter (Fig. 5a and Supplementary Fig. 5a). We have verified that the mCAR promoter-driven expression was specific for postnatal photoreceptors at P5, P10 and P16 (Supplementary Fig. 5b). We observed the scAAV-dependent accumulation of miR-183/96/182 already at P5, consistent with the failure of Ddx3x to repress T2-pri-miR-183/96/182 in early postnatal retina (Fig. 5b and Supplementary Fig. 5c). Most importantly, we found that premature accumulation of miR-183/96/182 processed from T2-pri-miR-183/96/182 resulted in a phenotype similar to that seen in the early knockdown of Ddx3x, with retinas at P10 and later having a wavy form with pronounced folds in the photoreceptor layer, as well as large regional variation in thickness (Fig. 5c,d and Supplementary Fig. 5d,e). Expression of T2-pri-miR-183/96/182 in mature retina, following AAV administration at P30, produced no changes in the organization of the photoreceptor layer at P46, despite a three- to fourfold increase in miR-183/96/182 expression (Supplementary Fig. 6).

To evaluate the properties of the T2-pri-miR-183/96/182-expressing retinas, we analysed rod- and cone-mediated photoresponses using a high-density microelectrode array at P16 (ref. 42). We observed spiking responses across seven logarithmic units of intensity (1–10^6^ photoisomerization per rod per second (R*/s)), including pure rod, rod–cone and pure cone-activating intensity ranges, suggesting proper activity of photoreceptors across a range of intensities (Supplementary Fig. 7).

We conclude that premature accumulation of miR-183/96/182, brought about either by Ddx3x knockdown or expression of constitutively processed T2-pri-miR-183/96/182, causes uneven distribution of cells within retinal photoreceptor and inner nuclear layers. This aberrant cellular organization appears to have no major effect on the activity of photoreceptors.

### Premature miR-183/96/182 accumulation inhibits Crb1 expression

We investigated the molecular mechanism underlying the photoreceptor layer phenotype induced by aberrant timing of miR-183/96/182 accumulation. Computational predictions identified Crb1 as a potential target of miR-183 and possibly also of miR-182 and miR-96, since they contain seed regions related to that of miR-183. At the apical site of the photoreceptor layer, an adhesion belt called an outer limiting membrane connects photoreceptors to Müller cells^6^. Notably, Crb proteins are components of the outer limiting membrane^6^, and *Crb1* gene knockout in mouse can cause photoreceptor layer changes similar to those we observed in retinas with precocious miR-183/96/182 accumulation^3^,^6^,^34^.

**Figure 6:**
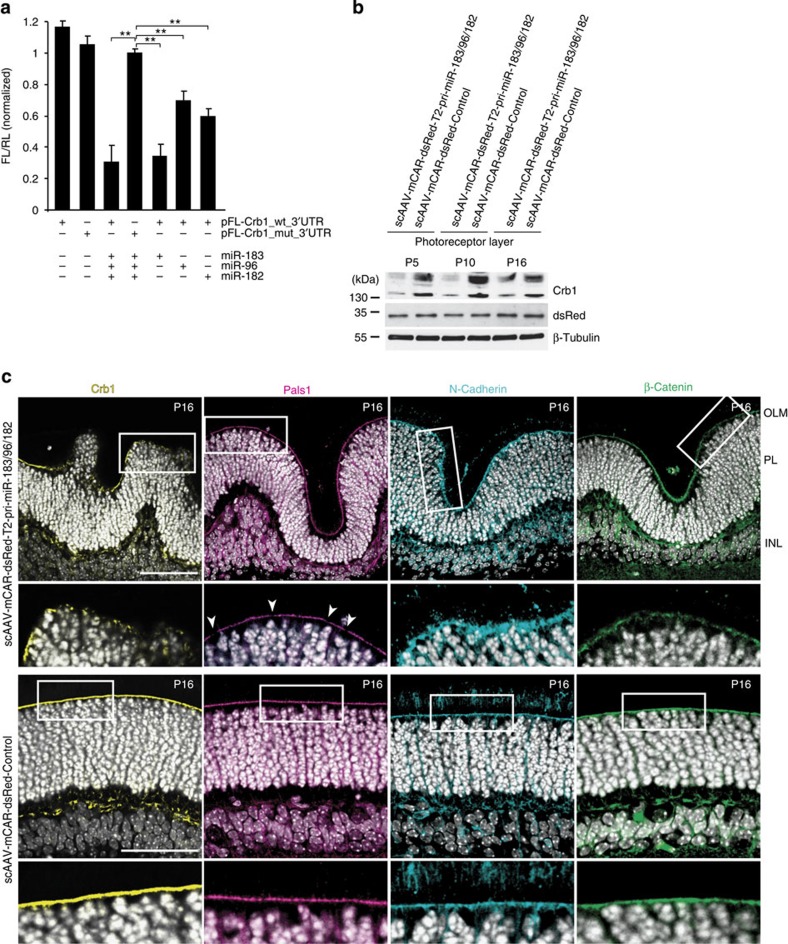
Crb1 is targeted by prematurely accumulating miR-183/96/182. (**a**) Activity of pFL-Crb1_wt_3′UTR and pFL-Crb1_mut_3′UTR reporters in HEK293T cells co-transfected with miRNA mimics. FL values, normalized to co-expressed RL, are means±s.e.m. *n*=5. **P<0.005; Mann–Whitney *U*-test. (**b**) Western blot analysis of Crb1, dsRed and β-Tubulin levels in laser-dissected photoreceptors of P5, P10 and P16 retinas infected with scAAV expressing T2-pri-miR-183/96/182 or control RNAs. For Crb1, two largest ∼153 and ∼142 kDa Crb1 isoforms are shown. (**c**) Confocal images of vertical sections of P16 T2-pri-miR-183/96/182 or control retinas stained with antibody against Crb1, Pals1, N-Cadherin and β-Catenin. Arrowheads indicate discontinuities in Pals1 staining. Hoechst-stained nuclei are in white. INL, inner nuclear layer; OLM, outer limiting membrane; PL, photoreceptor layer. Scale bar, 50 μm.

We asked whether Crb1 mRNA is a target of miR-183/96/182 repression. In HEK293T cells, the activity of a pFL-Crb1_wt_3′UTR reporter, bearing a wild-type Crb1 3′UTR, was significantly reduced by a co-transfected miR-183 mimic and less strongly reduced by miR-96 and miR-182 mimics compared with the reporter having mutations in the miR-183/96/182 sites (Fig. 6a). This suggested direct targeting of Crb1 mRNA by miR-183/96/182. More significantly, western blot analysis of protein extracts of retinas isolated from mice infected at P0 with scAAV-mCAR-dsRed-T2-pri-miR-183/96/182 revealed a marked decrease in Crb1 protein at P5, P10 and P16 compared with the controls (Fig. 6b). Levels of control proteins, including virus-encoded dsRed and endogenous β-tubulin, were unchanged. Furthermore, immunostaining of the P16 scAAV-mCAR-dsRed-T2-pri-miR-183/96/182-infected retina revealed a strong decrease in Crb1 expression in the outer limiting membrane compared with the control retina (Fig. 6c). Our finding that the mCAR promoter is only functional in photoreceptors (Supplementary Fig. 5b) is consistent with the expression of Crb1 being post-transcriptionally regulated by miR-183/96/182 in these cells. Since the photoreceptor-specific expression of miR-183/96/182 can control the level of Crb1 of the outer limiting membrane (Fig. 6c), it appears that in the early postnatal retina the photoreceptors rather than Müller cells^34^,^43^ represent a main source of Crb1 present in the membrane.

To determine whether integrity of the outer limiting membrane is modified by the precocious miR-183/96/182 accumulation, we examined by immunostaining the status of several proteins known to localize to the structure. This analysis revealed either discontinuities (staining for Pals1) or diffused patterns (staining for N-Cadherin and β-Catenin) in the scAAV-mCAR-dsRed-T2-pri-miR-183/96/182-infected retinas compared with controls (Fig. 6c). The observed changes in the distribution of these proteins are likely a consequence of the decreased Crb1 level.

Taken together, the data show that targeting of Crb1 mRNA by miR-183/96/182 prematurely accumulating in early postnatal photoreceptors results in a decrease in the Crb1 protein level, which affects integrity of the outer limiting membrane in the developing retina.

## Discussion

We have described a regulatory network consisting of lncRNA Rncr4, RNA helicase Ddx3x and miR-183/96/182 that controls the timing of miR-183/96/182 accumulation in photoreceptors. In this network, referred to as the ncRNA–Ddx3x network, Ddx3x inhibits processing of pri-miR-183/96/182 to mature miRNAs in early postnatal photoreceptors. This block is lifted by Rncr4, which antagonizes the repressive effect of Ddx3x. The network incorporates a feedback loop in which miR-183/96/182 targets Ddx3x mRNA. Perturbation of the network leads to large regional variation in the thickness of the photoreceptor layer, and likely secondarily, also of the adjacent inner nuclear layer. Below, we discuss possible mechanisms by which the three ncRNA–Ddx3x network components interact, and also a potential mechanism, which leads to the bulging of cells out from the retinal epithelium.

Rncr4 is encoded by a region upstream of pri-miR-183/96/182 and is transcribed in the opposite direction to pri-miR-183/96/182. The indirect influence of Rncr4 on the processing of pri-miR-183/96/182 by counteraction of the repressive effect of Ddx3x represents a novel form of gene expression control by lncRNAs. Other retina-specific lncRNAs share promoter-regulatory elements with genes encoding homeodomain transcription factors, including Six3, Pax6, Six6, Vax2, Otx2, Pax2 and Rx. These lncRNAs, however, operate in *cis* and regulate expression of their companion protein-coding genes via transcriptional facilitation or interference^10^,^11^,^35^. The tight control of miR-183/96/182 maturation in the retina by Rncr4 may be just one example of a more widespread lncRNA-dependent regulation of miRNA processing. In mice, a number of genes encoding non-retina-specific pri-miRNAs are adjacent to genes expressing lncRNAs (for examples, see Supplementary Table 1), suggesting that such ‘companion' lncRNAs may also control miRNA biogenesis in other tissues.

We found that Rncr4 has no influence on pri-miRNA processing in the absence of Ddx3x, indicating that the Rncr4 effect depends on the helicase-repressive function. The effect also requires that Ddx3x is catalytically proficient since mutations in its GKT and SAT motifs eliminated the pri-miR-183/96/182 repressor activity of the protein. Ddx3x is ubiquitously expressed and involved in various biological processes, including cell cycle progression and apoptosis, but also in cancer and the replication of human viruses^39^,^44^. Ddx3x catalyses rearrangements of the RNA structure and participates in many aspects of RNA function and metabolism, including mRNA splicing, translation and RNA transport^39^,^44^. Ddx3x has no apparent preferences for nucleotide sequence or composition but rather recognizes elements of RNA secondary structure^39^. Thus, Ddx3x binding may modify the structure of pri-miR-183/96/182 such that it is no longer efficiently recognized and processed by the Dgcr8/Drosha complex. It is well documented that a particular form of junction between the double-stranded pri-miRNA hairpin and its single-stranded RNA flanks is important for efficient and accurate processing by Dgcr8/Drosha^45^,^46^. However, the junction structures at the base of pri-miRNA hairpins are unlikely to be the main or only entry point for Ddx3x binding. Such junction structures are present in T2-pri-miR-183/96/182, the extensively deleted form of pri-miR-183/96/182. T2-pri-miR-183/96/182 processing to mature miRNAs is not a subject of Ddx3x-mediated repression, which suggests that elements other than junction structures within pri-miRNA hairpins are important for initiating Ddx3x binding. In this context, it is important to note that Ded1p, the yeast orthologue of Ddx3x, has the potential to bind RNA cooperatively, using multiple protomers for optimal RNA unwinding^39^,^47^. If Ddx3x functions in a similar way, this may explain why it affects processing of all three pri-miRNA hairpins present in pri-miR-183/96/182. Although Ddx3x can directly interact with different proteins^44^, given the observed specificity of regulation towards pri-miR-183/96/182, it is unlikely that Ddx3x binds to and modifies the Dgcr8/Drosha complex; such a situation would be expected to affect miRNA maturation more globally.

It is possible that Rncr4 interferes with the Ddx3x/pri-miR-183/96/182 interaction through base pairing or structural contacts with pri-miRNA. Recently, lncRNA *Uc.283+A* was shown to regulate miR-195 maturation by base pairing to pri-miR-195 and blocking its processing by Drosha^22^. We did not find extensive complementarity regions between Rncr4 and pri-miR-183/96/182, suggesting that the assumed interactions involve structural rather than sequence determinants. Alternatively, both RNAs may bind to Ddx3x in distinct ways, competing for its activity. There remains the possibility that additional factors participate in these regulatory events and that their activities are differentially affected by Ddx3x and Rncr4. Further work will establish the molecular basis of the controlled processing of pri-miR-183/96/182. It will also be interesting to find out whether, apart from being an miRNA source, the pri-miR-183/96/182 transcript has other functions that would justify its early P5 expression. Lumayag *et al*.^28^ identified spliced forms of pri-miR-183/96/182 with the potential to encode short polypeptides; however, their functions have not been investigated.

Why would the precocious accumulation of mature miR-183/96/182, in response to either Ddx3x knockdown or T2-pri-miR-183/96/182 expression, lead to variability in the thickness of the photoreceptor and inner nuclear layers? We suggest that Crb1, demonstrated here to be a target of miR-183/96/182 *in vitro* and *in vivo*, forms one of the links between the miRNA level and disruption of retinal architecture. Crb1 is known to be a component of the adhesion belt, in the outer limiting membrane, between Müller glia and photoreceptor cells^3^,^6^. Humans without Crb1 develop retinitis pigmentosa and Leber congenital amaurosis^48^,^49^,^50^. Most importantly, loss-of-function mutations in the *Crb1* gene are frequently associated with photoreceptor layer abnormalities^3^,^4^,^6^ similar to those we observed when mature miR-183/96/182 accumulated precociously. Significantly, we found that precocious formation of miR-183/96/182 not only results in a dramatic decrease in Crb1 but also, likely as a consequence of the Crb1 changes, in altered distribution of several other components of the outer limiting membrane, compromising its structural integrity (Fig. 6c).

**Figure 7:**
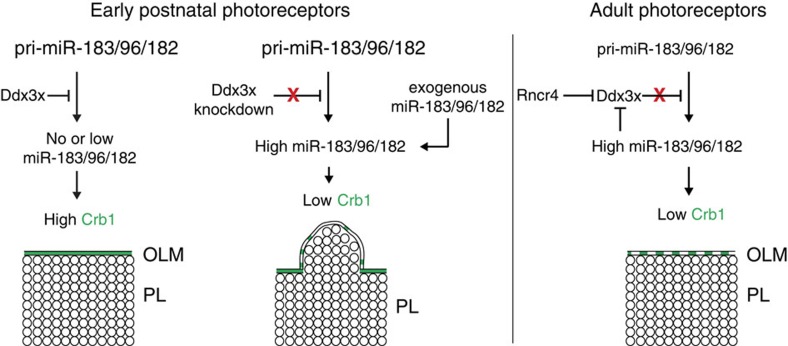
A model summarizing the ncRNA–Ddx3x network controlling photoreceptor layer architecture. In early postnatal photoreceptors, Ddx3x helicase inhibits processing of pri-miR-183/96/182 to mature miRNAs. Lack of miR-183/96/182 allows high Crb1 expression essential for establishment of a strong OLM that in turn ensures that photoreceptor cells form a uniform layer. The perturbation of the network by premature Ddx3x depletion or precocious accumulation of mature miR-183/96/182 decreases the Crb1 expression, resulting in weakening of OLM and, consequently, a large regional variation in the thickness of the photoreceptor layer and the adjacent inner nuclear layer (not shown in the scheme). In adults, Rncr4 antagonizes the repressive effect of Ddx3x on pri-miR-183/96/182 what leads to high miR-183/96/182 accumulation. In addition, miR-183/96/182 targets Ddx3x, possibly leading to further potentiation of pri-miR-183/96/182 processing. At that stage, organization of the photoreceptor layer is fully established and decreased expression of Crb1 and weakening of OLM may facilitate transport of molecules through the limiting membrane.

We postulate a model (Fig. 7) of how the developmentally timed regulation of miR-183/96/182 and its target Crb1 could contribute to uniform layer architecture in the retina. In early photoreceptors, when mature miR-183/96/182 is not present, Crb1 is expressed at a high level and ensures the formation of a strong adhesion belt between Müller glia and photoreceptors. During early postnatal development retinal cells continue to divide until ∼P6 (ref. 1) and the inner plexiform layer thickens until P15 (ref. 51), which together exert a force on retinal cells. The strong adhesion belt helps to keep retinal cells within a tight ‘bag' created by the inner and outer limiting membranes. After retina layers reach their final thickness, the continued sealing of the space where retinal cells reside may limit the diffusion of important molecules and, therefore, the adhesion belt gradually weakens. This weakening is due to the suppressed expression of Crb1 by miR-183/96/182, which slowly starts to accumulate after P5 in response to Rncr4. Precocious accumulation of mature miR-183/96/182 would lead to premature weakening of the outer limiting membrane barrier, resulting in a bag with a less robust wall. Groups of newly born cells would push against the weaker wall and bulge out to create large protrusions and, as a consequence, lead to the photoreceptor and inner nuclear layers of nonuniform thickness.

## Methods

### Animals

C57BL/6 mice obtained from Charles River (France) were bred in a pathogen-free environment with *ad libitum* access to food and drinking water. Age of mouse embryos and postnatal animals, of mixed sex, is indicated in individual experiments. All animal experiments and procedures were approved by the Swiss Veterinary Office.

### Laser-capture microdissection

Laser-capture microdissection of retina cell layers was performed as previously described^32^ with minor modifications. Briefly, isolated retinas were fixed in RCL2 reagent (Alphelys) on ice for 30 min, cryoprotected in 30% sucrose and embedded in Shandon M-1 (Thermo Fisher)-embedding matrix. Frozen tissues were cut into 20-μm-thick sections and mounted on RNase-free MMI (Molecular Machines & Industries AG) membrane slides. For RNA isolation, retinal sections were stained with haematoxylin (Molecular Machines & Industries AG) for 5 s, fixed and dehydrated for 50 s in 100% ethanol. After brief air-dry, cells from different retina cell layers were captured using MMI CellCut Plus System microscopy. For western blot analysis, sections were fixed and dehydrated in ice-cold methanol for 50 s. Once sections were briefly air-dried, the photoreceptor cell layer was captured and lysed in buffer containing 50 mM Tris (pH 7.5), 10 mM EDTA, 1% SDS and supplemented with protease inhibitor cocktail (Roche).

### RNA isolation

Total RNA from whole-mount retina or laser-dissected retina cell layers was extracted with the Arcturus PicoPure RNA Isolation Kit (Applied Biosystems) or Trizol reagent (Invitrogen). Total RNA from different mouse tissues were purchased from Ambion. Quantitative and qualitative RNA analyses were performed using a 2,100 Bioanalyzer (Agilent Technologies Inc.).

### RT–qPCR of miRNAs, pri-miRNAs and other cellular RNAs

Analysis of miRNA levels was performed using the Applied Biosystem Taqman microRNA Assay System (Applied Biosystems), as previously described^32^. Briefly, reverse transcription (RT) reactions containing 5 ng RNA, 1 × RT buffer, 0.25 mM each dNTP, 0.25 U μl^−1^ RNase inhibitor, 3.33 U μl^−1^ MultiScribe RT and 50 nM miRNA-specific RT primer were incubated for 30 min at 16 °C and 30 min at 42 °C. The 10-μl PCR reactions contained 0.67 μl of RT reaction, 1 × Taqman Universal PCR master mix and 1 μl of primers and a probe mix of the Taqman MicroRNA Assay. The reactions were incubated in a 48-well optical plate at 95 °C for 5 min, followed by 40 cycles of 95 °C for 15 s and 60 °C for 60 s. For pri-miRNAs and Rncr4 expression analysis, total RNA was reverse-transcribed using random hexamers and the Superscript III thermostabile RT system (Invitrogen) according to the manufacturer's instructions. RT–qPCR was performed using the Applied Biosystems StepOne System using standard protocol. Sequences of pri-miR-183, -96 and -182 primers used were described previously^32^. For Rncr4, forward (5′-ctcctgggaaatctcccttc-3′) and reverse (5′-gtgggcgtgtttcattcttt-3′) oligonucleotide primers were used. For small nuclear RNA U1 and brain cytoplasmic RNA BC1, forward (5′-tacttacctggcaggggagatac-3′ and 5′-gggttggggatttagctcag-3′) and reverse (5′-gaacgcagtcccccactac-3′ and 5′-aggttgtgtgtgccagttacc-3′) oligonucleotide primers were used, respecively. The threshold cycle (*C*_t_) values were determined using default threshold settings and expression fold change was calculated as 2-ΔΔ*C*_t_. Data were normalized to 18S rRNA (Figs 1b–f,h–i and 2a,g and Figs 5b, [Fig f5] and Supplementary Figs 1a,b,f and 3f, 5c, and 6a) or a control gene carried by pri-miR-183/96/182-expressing plasmids (Figs 1g, 2b,c and 3a and Supplementary Figs 2 and 4b).

### Subcellular RNA fractionation

Whole retina and the laser-captured photoreceptor layer were lysed on ice in a buffer containing 10 mM Tris (pH 7.4), 150 mM KCl and 8 mM MgAc supplemented with 0.5% Igepal CA-630 (Sigma). After lysate-filtering, the nuclei were separated from the cytoplasmic fraction by sedimentation through the sucrose (600 mM) cushion. RNA from the resulting nuclear and cytoplasm fractions was isolated with Trizol.

### Northern blot analysis

For Rncr4 detection, 10 μg of total RNA was resolved in a denaturing 1.2% (w/v) agarose gel and transferred to Hybond-N+ membrane using 10 × SSC. Rncr4-specific probe internally labelled with [α-^32^P] CTP was hybridized to RNA on the membrane in ULTRAhyb hybridization buffer (Ambion) at 65 °C. For U1a1 and BC1 ncRNA detection, the 5′ end labelled oligonucleotide probes (U1a1, 5′-5′-catccggagtgcaatggataagcctcgccctgggaaaaccaccttcgtgatcatggtatctcccctgccaggtaagtat-3′; BC1, ggttgtgtgtgccagttaccttgttt-3′) were used. Signals were detected using a PhosphorImager screen and a GE TyphoonTM 9400 scanner. RNA size was determined according to RNA molecular weight marker (Roche; Supplementary Fig. 8).

### Construction of miR-183/96/182-expressing vectors

To generate pEGP-L-pri-miR-183/96/182 and pEGP-T1-pri-miR-183/96/182 constructs, pri-miR-183/96/182 sequences corresponding to the chr6 30165145-30170699 (minus strand) for L-pri-miR-183/96/182 or chr6 30165358-30166397 fused to 30169118-30170157 (minus strand) for T1-pri-miR-183/96/182 were PCR-amplified using mouse retinal cDNA as a template and cloned into human β-globin intron of the pEGP-mir null backbone (Cell Biolabs) digested with XhoI (New England Biolabs). Truncated pri-miR-183/96/182 version 2 (T2) was designed *in silico* to retain pre-miR-183 and pre-miR-96 (chr6 30169427-30169777; minus strand) and pre-miR-182 (chr6 30165904-3030166007; minus strand) hairpins and some flanking sequences as annotated in miRBASE v.20 ( www.mirbase.org). The corresponding DNA was chemically synthesized by GENEWIZ (South Plainfield) and cloned into the XhoI-cleaved beta-globin intron of the pEGP backbone (to yield pEGP-T2-pri-miR-183/96/182) or into pCMV-MIR vector (OriGene Technologies) directly downstream of the CMV promoter (to yield pCMV-T2-pri-miR-183/96/182). Sequence of T2-pri-miR-183/96/182, with mature miRNAs underlined, is: 5′-cctctgcagggtctgcaggctggagagtgtgactcctgtcctgtgtatggcactggtagaattcactgtgaacagtctcagtcagtgaattaccgaagggccataaacagagcagagacagatccgcgagcaccttggagctcctcacccctttctgcctagacctctgtttccaggggtgccagggtacaaagacctcctctgctccttccccagagggcctgttccagtaccatctgcttggccgattttggcactagcacatttttgcttgtgtctctccgctgtgagcaatcatgtgtagtgccaatatgggaaaagcgggctgctgcggccacgttcacctcccccggcatcccataataaaaacaagtatgctggaggcctcccaccatttttggcaatggtagaactcacaccggtaaggtaatgggacccggtggttctagacttgccaactatggtgtaagtgctgagct-3′. To generate pCMV-L-pri-miR-183/96/182 and pCMV-pri-miR-128-1, L-pri-miR-183/96/182 and pri-miR-128-1 (chr1: 128202201–128202739) sequences were PCR-amplified from mouse cDNA and cloned into pCMV-MIR vector. Studies of pri-miR-183/96/182 processing in HEK293T cells were performed with both types of constructs, those containing pri-miRNA sequences cloned into human beta-globin intron of pEGP and those cloned into pCMV backbone, directly downstream of the CMV promoter. Similar results were obtained with both classes of constructs.

The scAAV-EF1a-GFP-T2-pri-miR-183/96/182 and scAAV-mCAR-dsRed-T2-pri-miR-183/96/182 constructs were obtained by cutting scAAV2-MCS (Cell Biolabs) with BalI/NotI. The transgene cassettes containing EF1a (sequence from pEGP-mmu-miR-182 plasmid; Cell Biolabs) or mCAR promoter^40^, EGFP (from pEGP-mmu-miR-182 plasmid; Cell Biolabs) or dsRed^52^, T2-pri-miR-183/96/182 sequence, WPRE motif and 5′-BalI and 3′-NotI adapters were chemically synthesized by GENEWIZ and inserted into the scAAV backbone. Control scAAV-EF1a-GFP-Control and scAAV-mCAR-dsRed-Control constructs contained fragments of β-globin intron (sequence from pEGP-mmu-miR-182 plasmid; Cell Biolabs) of the length corresponding to T2-pri-miR-183/96/182.

### Plasmids expressing DEAD-box RNA helicases

The pCMV6-AC plasmids expressing turbo GFP-tagged open reading frame (ORF) clones of DEAD-box polypeptide three X-linked (Ddx3x), polypeptide 5 (Ddx5) or polypeptide 17 (Ddx17) were purchased from OriGene Technologies. Control pCMV6-AC-GFP (OriGene Technologies) contained no additional fused ORF. The pCMV6-AC-Ddx3x_wt(siRes)-GFP construct resistant to two different siRNAs was created using a Quick-Change II XL Site-Directed Mutagenesis Kit (Stratagene), according to the manufacturer's protocol. The siRNA-targeted regions of Ddx3x mRNA: (I) 5′-GGAAATAGTCGCTGGTGTGAC-3′ and (II) 5′-GATTCCCTGACTCTAGTG-3′, encoding, respectively, GNSRWCD and DSLTLV peptide sequences, were replaced by 5′-GGAAATTCTAGATGGTGCGAC-3′ and 5′-GATAGTTTAACTTTAGTG-3′ sequences. Two siRNA-resistant constructs expressing Ddx3x mutants, pCMV6-AC-Ddx3x_mutK230E (siRes)-GFP and pCMV6-AC-Ddx3x_mutS382L (siRes)-GFP, were generated with site-directed mutagenesis using primers: mutK230E-F, 5′-caaacaggctctggagagactgcagcatttctcttgcccatct-3′; mutK230E-R, 5′-gagaaatgctgcagtctctccagagcctgtttgagcacaagcc-3′; mutS382L-F, 5′-cacactatgatgtttcttgctacttttcctaaggaaatacaga-3′; and mutS382L-R, 5′-cttaggaaaagtagcaagaaacatcatagtgtggcggacacct-3′.

### Plasmids expressing non-protein-coding RNAs

5′-RACE analysis, using a total RNA from the P28 retina and different reverse primers, identified a proximal transcription start site ∼500 bp upstream of the annotated Rncr4 5′-end. Sequence of the Rncr4, verified by 5′-RACE and 3′-RACE analyses, was PCR-amplified from retinal cDNA using oligonucleotide primers Rncr4-F (5′-tttgctggcccgccgagtg-3′) and Rncr4-R (5′-caaccaacttttgagtttaatttg-3′) and cloned into pCMV-MIR vector digested with SalI/XhoI. Sequences of Vax2OS1 (genebank NR_002873.2) and CrxOS1 (gene bank NM_001033638.2) were PCR-amplified from retinal cDNA using primers Vax2OS1-F (5′-actccttgtgtctgcggttc-3′), Vax2OS1-R (5′-tttattcaaaaagaaggatgcg-3′), CrxOS1-F (5′-atggaagcatctccacgttc-3′) and CrxOS1-R (5′-tcagaaatcttgtgacatgccatc-3′) as described previosly^10^. Obtained products were cloned into pCMV-MIR vector digested with BglII/EcoRV.

### AAV constructs expressing shRNAs and miRNA sponges

To generate scAAV-mCAR-dsRed-shDdx3x, the transgene cassettes containing the mCAR promoter, dsRed and pre-miRNA-like shRNAs either against mouse Ddx3x or containing a scrambled sequence folding into shRNA-like structure were chemically synthesized by GENEWIZ and inserted into the scAAV2-MCS backbone. The anti-Ddx3 shRNAs were designed according to BLOCK-iT RNAi Designer algorithm (Invitrogen) following the manufacturer's instructions. Sequences, with antisense sequence indicated in capitals, were as follows: Ddx3x-II (5′-AACACTAGAGTCAGGGAATCCgttttggccactgactgacggattcccactctagtgtt-3′); Ddx3x-I (5′-TCACACCAGCGACTATTTCCCgttttggccactgactgacgggaaatacgctggtgtga-3′). scAAV-mCAR-dsRed-shDdx3x-I and scAAV-mCAR-dsRed-shDdx3x-II were always co-injected together.

Triple sponge sequence specific for mouse miR-183/96/182 miRNAs was designed and obtained as described^32^. To generate AAV-mCAR-EGFP/triple sponge and AAV-mCAR-EGFP/control constructs, sequence corresponding to the Rho promoter region of the AAV2-Rho-EGFP/triple and AAV2-Rho-EGFP/control plasmids used previously^32^ was replaced by the mCAR promoter^40^ using proper restriction enzymes.

### Luciferase reporters

Renila luciferase (pRL) and firefly luciferase (pFL) vectors were as described^32^. pFL-Ddx3x_wt_3′UTR and pFL-Crb1_wt_3′UTR vectors, containing full-length 3′-UTRs of mouse Ddx3x (NM_010028.3) or Crb1 (NM_133239.2) mRNAs, respectively, were generated with RT–PCR using retinal cDNA as a template. The amplified fragments were cloned into the XbaI site present downstream of the FL ORF in pFL. Mutants containing three bases non-complementary to the miRNA seed region in each miRNA-binding site were created using a Quick-Change II XL Site-Directed Mutagenesis Kit according to the manufacturer's protocol.

### AAV production and delivery

AAVs were prepared by triple transfection of HEK293T cells using polyethylenimine with a plasmid bearing transgene sequence between the internal terminal repeats of AAV, the AAV-helper plasmid encoding Rep2 and Cap for serotype 8 and the pHGTI-Adeno1 plasmid harbouring helper adenoviral genes (provided by C.L. Cepko, Harvard Medical School, Boston, USA). For injection of viral particles, pups or adult mice were anaesthetized by controlled hypothermia or Ketamine/Xylazine intraperitoneal injection. A small incision was made with a sharp 30-gauge needle in the sclera near the lens. Overall, 0.5 μl (for pups) or 2 μl (for adults) of the AAV suspension were injected into the subretinal space using pulled-glass pipettes and a microinjector (Narishige, IM-11-2).

### Cell cultures and transfection

HEK293T cells were grown in DMEM (Invitrogen) supplemented with 2 mM L-glutamine and 10% heat-inactivated fetal calf serum at 37 °C in a humidified atmosphere containing 5% CO_2_. Cells were transfected with 200 ng of individual plasmids using the Nanofectin siRNA Kit, according to the manufacturer's instructions (PAA, Austria). For human Ddx3x knockdown, 20 nM of chemically synthesized (Sigma) siRNAs (I-s-5′-GAUUCACUGACCUUAGUGUdTdT-3′; I-as-5′-ACACUAAGGUCAGUGAAUCdTdT-3′; II-s-5′-GAAACAGUCGCUGGUGUGAdTdT-3′; II-as-5′-UCACACCAGCGACUGUUUCdTdT-3′) were used. In all experiments, siDdx3x-I and siDdx3x-II were co-transfected together.

### Western blot analysis

Cells were lysed on ice in a buffer containing 50 mM Tris pH 7.5, 10 mM dithiothreitol (DTT), 10 mM EDTA, 1% SDS and 1 × complete protease inhibitor cocktail (Roche). Protein lysates were separated using SDS–PAGE followed by electrotransfer to the polyvinylidene difluoride membrane (Millipore). Membranes were incubated for 2 h at room temperature with primary and then with secondary antibodies. Primary antibodies were as follows: rabbit-anti-Ddx3x (1:500, Millipore), mouse-anti-β-Tubulin (1:10,000; Sigma), rabbit-anti-RFP (1:500, Rockland), rabbit-anti-Drosha (1:100, Upstate), rabbit-anti-Dgcr8 (1:200, Abcam), rabbit-anti-Dicer D347 (1:500)^53^ and goat-anti-Crb1 (1:100, Santa Cruz). IRDye680 or IRDye800CW secondary antibodies were used and membranes were analysed with the Odyssey Infrared Imaging System (LI-COR Biosciences). Protein ladder PageRulerTM Plus (Fermentas) was used as a protein molecular weight marker (Supplementary Fig. 8).

### Immunohistochemistry

Retinas were fixed for 30 min in 4% (wt/vol) paraformaldehyde in PBS and washed with PBS for 24 h at 4 °C. To improve antibody penetration, retinas were subjected to freeze thaw cycles after cryoprotection with 30% (wt/vol) sucrose. After washing in PBS, retinal whole mounts or 3% agarose-embedded (SeaKem Le Agarose, Lonza) 100-μm-thick vibratome sections (Leica VT1000S vibratome) were incubated for 2 h in blocking buffer containing 10% (vol/vol) normal donkey serum (Chemicon), 1% (wt/vol) bovine serum albumin (BSA), 0.5% (vol/vol) Triton X-100 and 0.01% sodium azide (Sigma) in PBS. Primary antibody treatment was performed for 5 days at room temperature in buffer containing 3% (vol/vol) NDS, 1% (wt/vol) BSA, 0.01% (wt/vol) sodium azide and 0.5% Triton X-100 in PBS. Secondary antibody incubation was performed for 2 h at room temperature in buffer supplemented with Hoechst (Molecular Probes; 10 μg ml^−1^). After wash in PBS, retinas were embedded in Prolong Gold antifade (Molecular Probes). The following sets of primary and secondary antibodies were used: rat-anti-GFP (1:400, Nacalai Tesque), mouse-anti-Rho (1:200, Sigma), goat anti-ChAT (1:200, Chemicon), rabbit-anti-mCAR (1:200, Millipore), mouse-anti-PKC (1:200, BD Bioscience Pharmingen), mouse-anti-GS (1:200, Chemicon), goat-anti-OPN1MW (1:200, Santa Cruz), goat-anti-OPN1SW (1:200, Santa Cruz), rat-anti-RFP (1:500, ChromoTek), goat-anti-Crb1 (1:50, Santa Cruz), rabbit-anti-Pals1 (1:200, Millipore), mouse-anti-N-Cadherin (1:200, BD Transduction Laboratories), mouse-anti-β-Catenin (1:200, BD Transduction Laboratories), donkey anti-rat IgG conjugated with Alexa Fluor 488 (1:200, Invitrogen), donkey anti-rabbit IgG conjugated with Alexa Fluor 488, 555 or 633 (1:200, Invitrogen), donkey anti-mouse IgG conjugated with Alexa Fluor 488, 555 or 647 (1:200, Invitrogen), donkey anti-goat IgG conjugated with Alexa Fluor 633 (1:200, Invitrogen).

### Confocal microscopy

Zeiss LSM 700 laser scanning confocal microscope was used to acquire images of antibody-stained retinas with EC Plan-Neofluar × 40 or × 60 oil objectives. Images were processed using Imaris (Bitplane) or ImageJ 1.47u (NIH).

### RNA pull-down assay

For RNA pull-down assay, L-pri-miR-183/96/182 and pri-miR-128-1 RNAs were *in vitro* transcribed using the pCMV backbone constructs as templates and MEGAscript T7 kit (Ambion). RNAs were 3′-end-biotinylated using the Pierce RNA 3′ End Biotinylation Kit (Thermo Scientific) according to the manufacturer's instructions. *In vitro* transcripts were analysed on agarose gels and purified according to the manufacturer's instructions (Ambion). Cell lysate was prepared by incubating isolated retinas in buffer containing 50 mM Tris-HCl pH 7.5, 150 mM KCl, 2 mM DTT, 10% glycerol, 0.5% Triton X-100, 100 U ml^−1^ of RNase inhibitor supplemented with protease inhibitor (Roche). Biotinylated RNA transcripts (2 μg) and cell lysate (300 μg) were incubated for 30 min at room temperature in buffer containing 20 mM Tris-HCl pH 7.5, 100 mM KCl and 2 mM EDTA. Then, 100 μl of streptavidin-conjugated magnetic beads (Roche) saturated with tRNA was added to the extract for 15 min at room temperature. After three washes with buffer containing 20 mM Tris-HCl pH 8.0, 300 mM KCl and 0.2 mM EDTA, bound proteins were eluted and subsequently analysed using western blot analysis.

### Reporter luciferase assays

HEK293T cells were co-transfected with pFL reporters, control pRL plasmid and miRNA mimics using the Nanofectin siRNA Kit, according to the manufacturer's instructions (PAA). Cell lysates were prepared with Passive Lysis Buffer (Promega) 48 h after transfection and luciferase activities were measured using the Dual Luciferase Reporter Assay (Promega).

### RNA immunoprecipitation assay

RNA immunoprecipitation was performed by incubating 30 μg of anti-Ddx3x antibody or isotypic IgGs from rabbit serum (Sigma) with 40 μl of protein A Sepharose beads (GE Healthcare Life Sciences) for 2 h at 4 °C. Retinal cells were lysed in buffer containing 25 mM Tris-HCl pH 7.5, 150 mM NaCl, 2 mM DTT, 10% glycerol, 0.5% Triton X-100 and 10 U ml^−1^ of RNase inhibitor supplemented with protease inhibitor (Roche). Samples were preincubated for 1 h at 4 °C with 30 μl of beads. The supernatant was added to antibody-coated beads and incubated overnight at 4 °C. Beads were washed four times with buffer containing 50 mM Tris-HCl pH 7.5, 300 mM NaCl, 2 mM DTT and 0.05% Triton X-100, and split for protein and RNA analysis.

### Multielectrode array recordings

Animals were dark-adapted for 2 h before retina isolation. Eyes were dissected under infrared illumination (FEL0750—Longpass Filter, cuton wavelength: 750 nm Thorlabs) using night vision device (NVM-01-2-HR-A, www.tmt.ch). Recording was performed using microelectronics-based microelectrode arrays having 11,011 platinum electrodes with diameters of 7 μm and electrode centre-to-centre distances of 18 μm over an area of 2 × 1.75 mm^2^ (refs 42, 54). Extracellular action potentials were recorded at high temporal resolution (20 kHz) and with low noise levels (∼7–9 μV_rms_). To evoke light responses we used moving rectangular 1 × 0.5 mm^2^ bar, background illumination 2 μW cm^−2^ and stimulus irradiance 470 μW cm^−2^. To measure rod responses, neutral density filters (NE10B-NE60B, Thorlabs) were used. Isolated spike trains from single retinal ganglion cells were convolved with a Gaussian kernel (sigma=50 ms). The peak response of convolved spike trains was used for quantifying light responses.

### Statistical analysis

The Mann–Whitney *U*-test was used to compare data. Significance levels are indicated by *P<0.05, **P<0.01 and ***P<0.001. n.s. (not significant) means *P*≥0.05. The error bars represent s.e.m.

## Additional information

**How to cite this article:** Krol, J. *et al*. A network comprising short and long noncoding RNAs and RNA helicase controls mouse retina architecture. *Nat. Commun.* 6:7305 doi: 10.1038/ncomms8305 (2015).

## Supplementary Material

Supplementary Information Supplementary Figures 1-8, Supplementary Tables 1

## References

[b1] Young R. W. Cell differentiation in the retina of the mouse. Anat. Rec. 212, 199–205 (1985). 3842042 10.1002/ar.1092120215

[b2] Cepko C. L., Austin C. P., Yang X., Alexiades M. & Ezzeddine D. Cell fate determination in the vertebrate retina. Proc. Natl Acad. Sci. USA 93, 589–595 (1996). 8570600 10.1073/pnas.93.2.589 PMC40096

[b3] Mehalow A. K. . CRB1 is essential for external limiting membrane integrity and photoreceptor morphogenesis in the mammalian retina. Hum. Mol. Genet. 12, 2179–2189 (2003). 12915475 10.1093/hmg/ddg232

[b4] van de Pavert S. A. . Crumbs homologue 1 is required for maintenance of photoreceptor cell polarization and adhesion during light exposure. J. Cell Sci. 117, 4169–4177 (2004). 15316081 10.1242/jcs.01301

[b5] Koike C. . Function of atypical protein kinase *C lambda* in differentiating photoreceptors is required for proper lamination of mouse retina. J. Neurosci. 25, 10290–10298 (2005). 16267237 10.1523/JNEUROSCI.3657-05.2005 PMC6725782

[b6] Alves C. H., Pellissier L. P. & Wijnholds J. The CRB1 and adherens junction complex proteins in retinal development and maintenance. Prog. Retin. Eye Res. 40, 35–52 (2014). 24508727 10.1016/j.preteyeres.2014.01.001

[b7] Haider N. B., Naggert J. K. & Nishina P. M. Excess cone cell proliferation due to lack of a functional NR2E3 causes retinal dysplasia and degeneration in rd7/rd7 mice. Hum. Mol. Genet. 10, 1619–1626 (2001). 11487564 10.1093/hmg/10.16.1619

[b8] Mears A. J. . Nrl is required for rod photoreceptor development. Nat. Genet. 29, 447–452 (2001). 11694879 10.1038/ng774

[b9] Fatica A. & Bozzoni I. Long non-coding RNAs: new players in cell differentiation and development. Nat. Rev. Genet. 15, 7–21 (2014). 24296535 10.1038/nrg3606

[b10] Alfano G. . Natural antisense transcripts associated with genes involved in eye development. Hum. Mol. Genet. 14, 913–923 (2005). 15703187 10.1093/hmg/ddi084

[b11] Rapicavoli N. A. & Blackshaw S. New meaning in the message: noncoding RNAs and their role in retinal development. Dev. Dyn. 238, 2103–2114 (2009). 19191220 10.1002/dvdy.21844

[b12] Rapicavoli N. A., Poth E. M. & Blackshaw S. The long noncoding RNA RNCR2 directs mouse retinal cell specification. BMC Dev. Biol. 10, 49 (2010). 20459797 10.1186/1471-213X-10-49 PMC2876091

[b13] Rapicavoli N. A., Poth E. M., Zhu H. & Blackshaw S. The long noncoding RNA Six3OS acts in trans to regulate retinal development by modulating Six3 activity. Neural Dev. 6, 32 (2011). 21936910 10.1186/1749-8104-6-32 PMC3191369

[b14] Young T. L., Matsuda T. & Cepko C. L. The noncoding RNA taurine upregulated gene 1 is required for differentiation of the murine retina. Curr. Biol. 15, 501–512 (2005). 15797018 10.1016/j.cub.2005.02.027

[b15] Meola N., Pizzo M., Alfano G., Surace E. M. & Banfi S. The long noncoding RNA Vax2os1 controls the cell cycle progression of photoreceptor progenitors in the mouse retina. RNA 18, 111–123 (2012). 22128341 10.1261/rna.029454.111 PMC3261733

[b16] Fabian M. R., Sonenberg N. & Filipowicz W. Regulation of mRNA translation and stability by microRNAs. Annu. Rev. Biochem. 79, 351–379 (2010). 20533884 10.1146/annurev-biochem-060308-103103

[b17] Kim V. N., Han J. & Siomi M. C. Biogenesis of small RNAs in animals. Nat. Rev. Mol. Cell Biol. 10, 126–139 (2009). 19165215 10.1038/nrm2632

[b18] Krol J., Loedige I. & Filipowicz W. The widespread regulation of microRNA biogenesis, function and decay. Nat. Rev. Genet. 11, 597–610 (2010). 20661255 10.1038/nrg2843

[b19] Cesana M. . A long noncoding RNA controls muscle differentiation by functioning as a competing endogenous RNA. Cell 147, 358–369 (2011). 22000014 10.1016/j.cell.2011.09.028 PMC3234495

[b20] Wang Y. . Endogenous miRNA sponge lincRNA-RoR regulates Oct4, Nanog, and Sox2 in human embryonic stem cell self-renewal. Dev. Cell 25, 69–80 (2013). 23541921 10.1016/j.devcel.2013.03.002

[b21] Kallen A. N. . The imprinted H19 lncRNA antagonizes let-7 microRNAs. Mol. Cell 52, 101–112 (2013). 24055342 10.1016/j.molcel.2013.08.027 PMC3843377

[b22] Liz J. . Regulation of pri-miRNA processing by a long noncoding RNA transcribed from an ultraconserved region. Mol. Cell 55, 138–147 (2014). 24910097 10.1016/j.molcel.2014.05.005

[b23] Ryan D. G., Oliveira-Fernandes M. & Lavker R. M. MicroRNAs of the mammalian eye display distinct and overlapping tissue specificity. Mol. Vis. 12, 1175–1184 (2006). 17102797

[b24] Karali M., Peluso I., Marigo V. & Banfi S. Identification and characterization of microRNAs expressed in the mouse eye. Invest. Ophthalmol. Vis. Sci. 48, 509–515 (2007). 17251443 10.1167/iovs.06-0866

[b25] Arora A. . Prediction of microRNAs affecting mRNA expression during retinal development. BMC Dev. Biol. 10, 1 (2010). 20053268 10.1186/1471-213X-10-1 PMC2821300

[b26] Damiani D. . Dicer inactivation leads to progressive functional and structural degeneration of the mouse retina. J. Neurosci. 28, 4878–4887 (2008). 18463241 10.1523/JNEUROSCI.0828-08.2008 PMC3325486

[b27] Georgi S. A. & Reh T. A. Dicer is required for the transition from early to late progenitor state in the developing mouse retina. J. Neurosci. 30, 4048–4061 (2010). 20237275 10.1523/JNEUROSCI.4982-09.2010 PMC2853880

[b28] Lumayag S. . Inactivation of the microRNA-183/96/182 cluster results in syndromic retinal degeneration. Proc. Natl. Acad. Sci. USA 110, E507–E516 (2013). 23341629 10.1073/pnas.1212655110 PMC3568372

[b29] Zhu Q. . Sponge transgenic mouse model reveals important roles for the microRNA-183 (miR-183)/96/182 cluster in postmitotic photoreceptors of the retina. J. Biol. Chem. 286, 31749–31760 (2011). 21768104 10.1074/jbc.M111.259028 PMC3173082

[b30] Sanuki R. . miR-124a is required for hippocampal axogenesis and retinal cone survival through Lhx2 suppression. Nat. Neurosci. 14, 1125–1134 (2011). 21857657 10.1038/nn.2897

[b31] Xu S., Witmer P. D., Lumayag S., Kovacs B. & Valle D. MicroRNA (miRNA) transcriptome of mouse retina and identification of a sensory organ-specific miRNA cluster. J. Biol. Chem. 282, 25053–25066 (2007). 17597072 10.1074/jbc.M700501200

[b32] Krol J. . Characterizing light-regulated retinal microRNAs reveals rapid turnover as a common property of neuronal microRNAs. Cell 141, 618–631 (2010). 20478254 10.1016/j.cell.2010.03.039

[b33] Busskamp V. . miRNAs 182 and 183 are necessary to maintain adult cone photoreceptor outer segments and visual function. Neuron 83, 586–600 (2014). 25002228 10.1016/j.neuron.2014.06.020

[b34] van Rossum A. G. . Pals1/Mpp5 is required for correct localization of Crb1 at the subapical region in polarized Muller glia cells. Hum. Mol. Genet. 15, 2659–2672 (2006). 16885194 10.1093/hmg/ddl194

[b35] Blackshaw S. . Genomic analysis of mouse retinal development. PLoS Biol. 2, E247 (2004). 15226823 10.1371/journal.pbio.0020247 PMC439783

[b36] Hansen G. M. . Large-scale gene trapping in C57BL/6N mouse embryonic stem cells. Genome Res. 18, 1670–1679 (2008). 18799693 10.1101/gr.078352.108 PMC2556270

[b37] Gregory R. I. . The Microprocessor complex mediates the genesis of microRNAs. Nature 432, 235–240 (2004). 15531877 10.1038/nature03120

[b38] Soto-Rifo R. . DEAD-box protein DDX3 associates with eIF4F to promote translation of selected mRNAs. EMBO J. 31, 3745–3756 (2012). 22872150 10.1038/emboj.2012.220 PMC3442272

[b39] Sharma D. & Jankowsky E. The Ded1/DDX3 subfamily of DEAD-box RNA helicases. Crit. Rev. Biochem. Mol. Biol. 49, 343–360 (2014). 25039764 10.3109/10409238.2014.931339

[b40] Busskamp V. . Genetic reactivation of cone photoreceptors restores visual responses in retinitis pigmentosa. Science 329, 413–417 (2010). 20576849 10.1126/science.1190897

[b41] McCarty D. M. Self-complementary AAV vectors; advances and applications. Mol. Ther. 16, 1648–1656 (2008). 18682697 10.1038/mt.2008.171

[b42] Fiscella M. . Recording from defined populations of retinal ganglion cells using a high-density CMOS-integrated microelectrode array with real-time switchable electrode selection. J. Neurosci. Methods 211, 103–113 (2012). 22939921 10.1016/j.jneumeth.2012.08.017 PMC5419501

[b43] den Hollander A. I. . Isolation of Crb1, a mouse homologue of Drosophila crumbs, and analysis of its expression pattern in eye and brain. Mech. Dev. 110, 203–207 (2002). 11744384 10.1016/s0925-4773(01)00568-8

[b44] Soto-Rifo R. & Ohlmann T. The role of the DEAD-box RNA helicase DDX3 in mRNA metabolism. Wiley Interdiscip. Rev. RNA 4, 369–385 (2013). 23606618 10.1002/wrna.1165

[b45] Han J. . Molecular basis for the recognition of primary microRNAs by the Drosha-DGCR8 complex. Cell 125, 887–901 (2006). 16751099 10.1016/j.cell.2006.03.043

[b46] Ma H., Wu Y., Choi J. G. & Wu H. Lower and upper stem-single-stranded RNA junctions together determine the Drosha cleavage site. Proc. Natl Acad. Sci. USA 110, 20687–20692 (2013). 24297910 10.1073/pnas.1311639110 PMC3870748

[b47] Yang Q., Del Campo M., Lambowitz A. M. & Jankowsky E. DEAD-box proteins unwind duplexes by local strand separation. Mol. Cell 28, 253–263 (2007). 17964264 10.1016/j.molcel.2007.08.016

[b48] den Hollander A. I. . Mutations in a human homologue of Drosophila crumbs cause retinitis pigmentosa (RP12). Nat. Genet. 23, 217–221 (1999). 10508521 10.1038/13848

[b49] den Hollander A. I. . Leber congenital amaurosis and retinitis pigmentosa with Coats-like exudative vasculopathy are associated with mutations in the crumbs homologue 1 (CRB1) gene. Am. J. Hum. Genet. 69, 198–203 (2001). 11389483 10.1086/321263 PMC1226034

[b50] Lotery A. J. . Mutations in the CRB1 gene cause Leber congenital amaurosis. Arch. Ophthalol. 119, 415–420 (2001). 10.1001/archopht.119.3.415 11231775

[b51] Fisher L. J. Development of synaptic arrays in the inner plexiform layer of neonatal mouse retina. J. Comp. Neurol. 187, 359–372 (1979). 489784 10.1002/cne.901870207

[b52] Matsuda T. & Cepko C. L. Electroporation and RNA interference in the rodent retina *in vivo* and *in vitro*. Proc. Natl Acad. Sci. USA 101, 16–22 (2004). 14603031 10.1073/pnas.2235688100 PMC314130

[b53] Billy E., Brondani V., Zhang H., Muller U. & Filipowicz W. Specific interference with gene expression induced by long, double-stranded RNA in mouse embryonal teratocarcinoma cell lines. Proc. Natl Acad. Sci. USA 98, 14428–14433 (2001). 11724966 10.1073/pnas.261562698 PMC64698

[b54] Frey U., Egert U., Heer F., Hafizovic S. & Hierlemann A. Microelectronic system for high-resolution mapping of extracellular electric fields applied to brain slices. Biosens. Bioelectron. 24, 2191–2198 (2009). 19157842 10.1016/j.bios.2008.11.028

