# Supplementary material for: A network comprising short and long noncoding RNAs and RNA helicase controls mouse retina architecture
Source: Nat Commun. 2015 Jun 4;6:7305. doi: 10.1038/ncomms8305 (PMC4468907; doi:10.1038/ncomms8305)
Supplement: Supplementary Information — Supplementary Figures 1-8, Supplementary Tables 1 [file ncomms8305-s1.pdf]

SUPPLEMENTARY INFORMATION

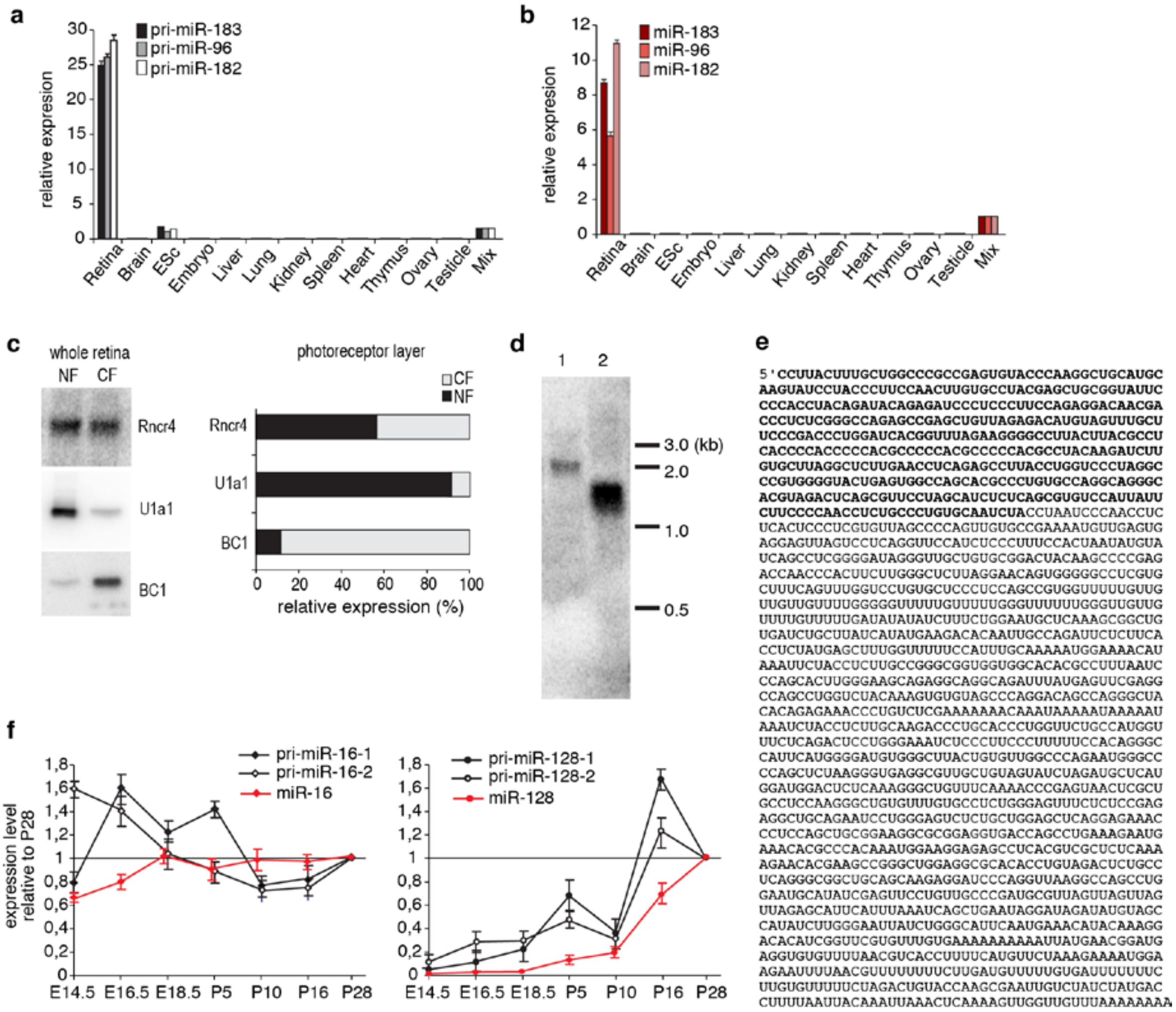

Supplementary Figure 1 | Characterization of miR-183/96/182 and Rncr4 expression in retina (a-d) and evidence that delay in accumulation of mature miRNAs is specific to pri-miR-183/96/182.

(**a, b**) RT-qPCR analysis of pri-miR-183/96/182 and miR-183/96/182 levels shows enriched expression in the retina when compared to other mouse tissues. Values, normalized to values for the mixture (Mix) of RNAs from different tissues (set to 1), are means ± s.e.m. *n* = 3. (**c**) Northern (left panel) and RT-qPCR (right panel) analyses of nuclear (NF) and cytoplasmic (CF) RNA fractions of whole retina (left panel) or photoreceptor layer (right panel) reveal subcellular localization of Rncr4. U1a1 snRNA and brain cytoplasmic ncRNA 1 (BC1), having nuclear and cytoplasmic localization, respectively, were used as controls. (**d**) Northern analysis detects Rncr4 as a ~1.9 kb transcript in P28 retina (lane 1) and as 1.4 kb transcript when RNA is ectopically expressed in HEK293T cells from plasmid carrying GenBank annotated Rncr4 sequence (lane 2). (**e**) Rncr4 sequence confirmed by 5’- and 3’- RACE. The additional 5’-

terminal experimentally validated sequence of Rncr4 is in bold. (**f**) RT-qPCR analysis of mature miR-128 and miR-16 and their pri-miRNAs (note that each of the miRNAs is encoded by two different genes) in developing retina shows lack of the processing delay similar to that seen for miR-183/96/182. Levels of pri-miRNAs (black) and mature miRNAs (red) are normalized to values at P28, which are set to 1. Normalized values are means ± s.e.m. n = 10 retinas per group.

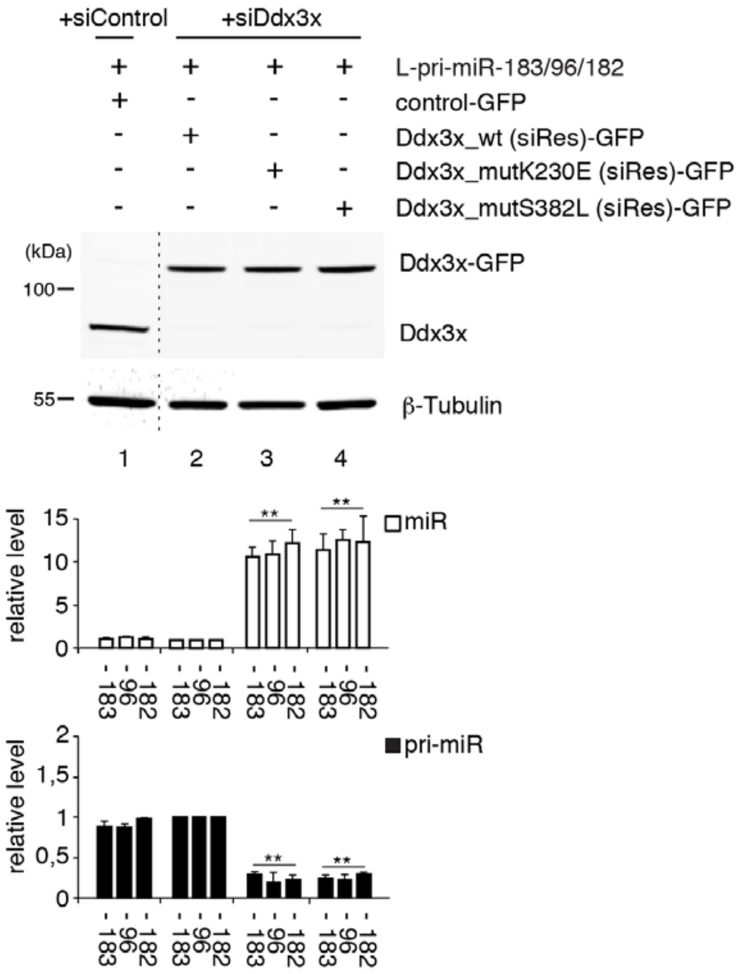

Supplementary Figure 2 | Inhibition of the pri-miR-183/96/182 processing by Ddx3x depends on its ATPase and helicase activities.

HEK293T cells treated with control or Ddx3x-specific siRNA were cotransfected with plasmids expressing L-pri-miR-183/96/182 and either control or the siRNA resistant (siRes) wild type (wt) or mutant Ddx3x. Western blots show levels of ectopically expressed and endogenous proteins. Pri-miR-183/96/182 and miR-183/96/182 levels were analyzed by RT-qPCR. Mutations in ATPase (MutK230E) and helicase (MutS383L) motifs abolished inhibitory effect of Ddx3x on pri-miR-183/96/182 processing. Normalized values (siRes wt Ddx3x is set to 1; lane 2) represent means ± s.e.m. $n = 5$. $^{**}P < 0.01$; Mann-Whitney U-test.

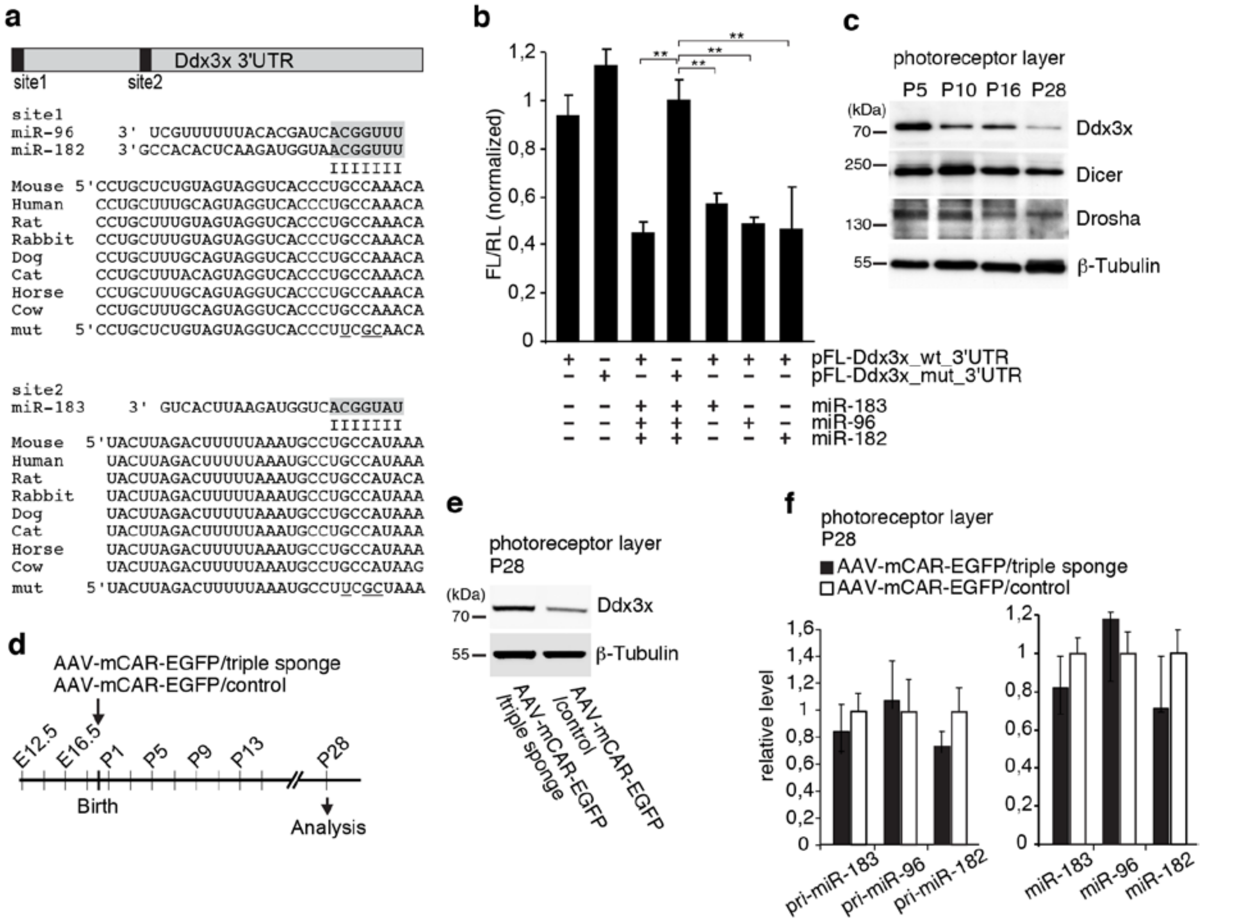

Supplementary Figure 3 | Ddx3x is a miR-183/96/182 target.

(**a**) Sequence conservation and base pairing of miR-183/96/182 seed sequences to Ddx3x 3'UTR. All three miRNAs have related seed regions and thus can target the same mRNA. Residues mutated in the miRNA seed regions of the mutant reporter are underlined. (**b**) Activity of pFL-Ddx3x_wt_3'UTR and pFL-Ddx3x_mut_3'UTR reporters in HEK293T cells cotransfected with miRNA mimics. FL values, normalized to coexpressed RL, are means ± s.e.m. $n = 5$. $^{**}P < 0.005$; Mann-Whitney U-test. (**c**) Western analysis of Ddx3x, Dicer, Drosha, and β-Tubulin in the laser-dissected photoreceptors at P5, P10, P16, and P28. (**d**) Schematic representation of experiments shown in panels e and f. Eyes were injected subretinally at P0 with AAV2-mCAR-EGFP-triple sponge or control virus. (**e**) Western analysis reveals increase of Ddx3x level in the laser-dissected P28 photoreceptors infected with AAV2-mCAR-EGFP-triple sponge when compared to control. (f) RT-qPCR analysis of miR-183/96/182 and pri-miR-183/96/182 levels in photoreceptors of P28 retinas infected with AAV2-mCAR-EGFP-triple sponge or control virus. Values, normalized to controls set at 1, are means ± s.e.m. $n = 3$.

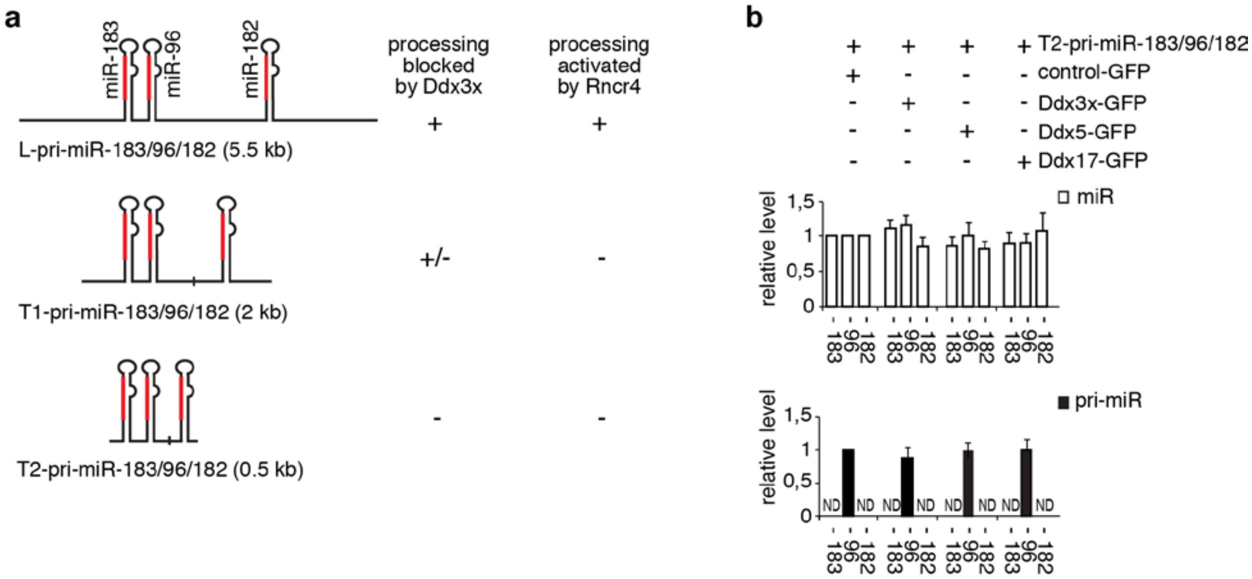

Supplementary Figure 4 | Truncated pri-miR-183/96/182 (T2 form) is not a subject of Ddx3x repression.

(**a**) Overview of experimental results. L-, T1-, and T2-pri-miR-183/96/182 forms are schematically shown on the left (for more details, see Methods). In HEK293T cells, depleted of endogenous Ddx3x, processing of T1-pri-miR-183/96/182 is weakly inhibited whereas T2-pri-miR-183/96/182 is not inhibited by the ectopically expressed siRes Ddx3x. (**b**) RT-qPCR analysis of miR-183/96/182 and pri-miR-183/96/182 levels in HEK293T cells expressing T2-pri-miR-183/96/182 and GFP fusions of different helicases (Ddx3x, Ddx5, or Ddx17) or GFP used as a control. Note that only pri-miR-96, but not two other pri-miRNAs, was detectable by RT-qPCR. Normalized values are means ± s.e.m. $n = 3$. ND, not detectable.

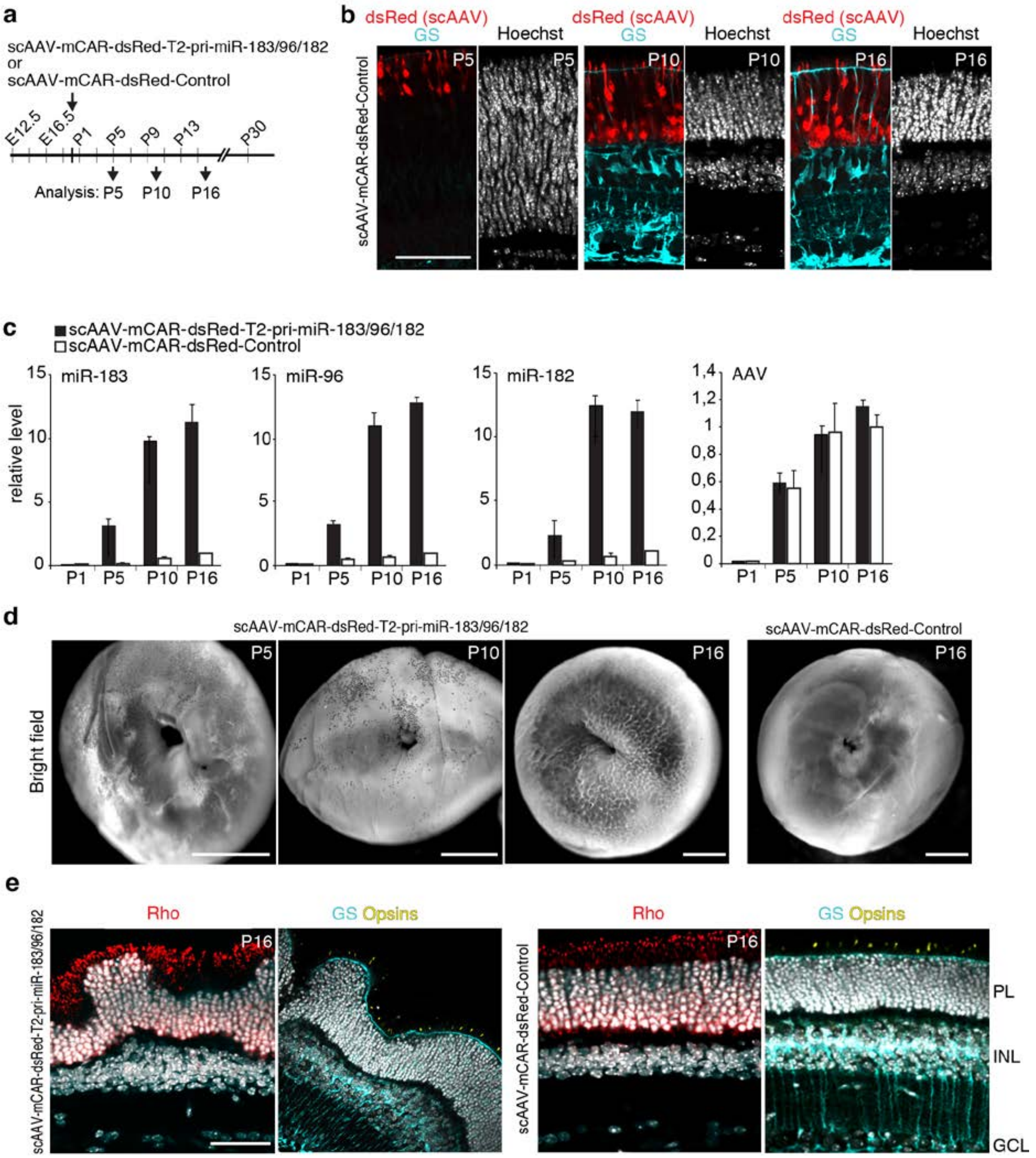

Supplementary Figure 5 | Precocious accumulation of miR-183/96/182 in photoreceptors affects layer organization of the retina.

(**a**) Schematic representation of the experiment. Eyes were injected subretinally at P0 with scAAV expressing T2-pri-miR-183/96/182 or control RNA under the photoreceptor-specific mCAR promoter. (**b**) Verification that mCAR promoter is active in both early and late postnatal photoreceptors but not in Müller cells. Confocal images of vertical sections of P5, P10, and P16 retinas are shown. Photoreceptors and glia are visualized by staining with antibodies against dsRed (expression of which is driven by mCAR promoter) and glutamine synthetase (GS), respectively. (**c**) RT-qPCR analysis of miR-183/96/182 and AAV levels in T2-

pri-miR-183/96/182 and control retinas. Values, normalized to values of control at P16 are means ± s.e.m. $n = 3$. (**d**) Bright field images of P5, P10, and P16 retinas expressing T2-pri-miR-183/96/182, and P16 retina expressing control RNA. (**e**) Confocal images of vertical sections of the P16 retinas expressing either T2-pri-miR-183/96/182 or control RNA. Retinas were stained with antibodies against rhodopsin (Rho), glutamine synthetase (GS) or opsins to visualize rods, Müller cells, and cones respectively. Hoechst stained nuclei are in white. PL, photoreceptor layer; INL, inner nuclear layer; GCL, ganglion cell layer. Scale bars: (**d**) 500 μm; (b, **e**) 50 μm.

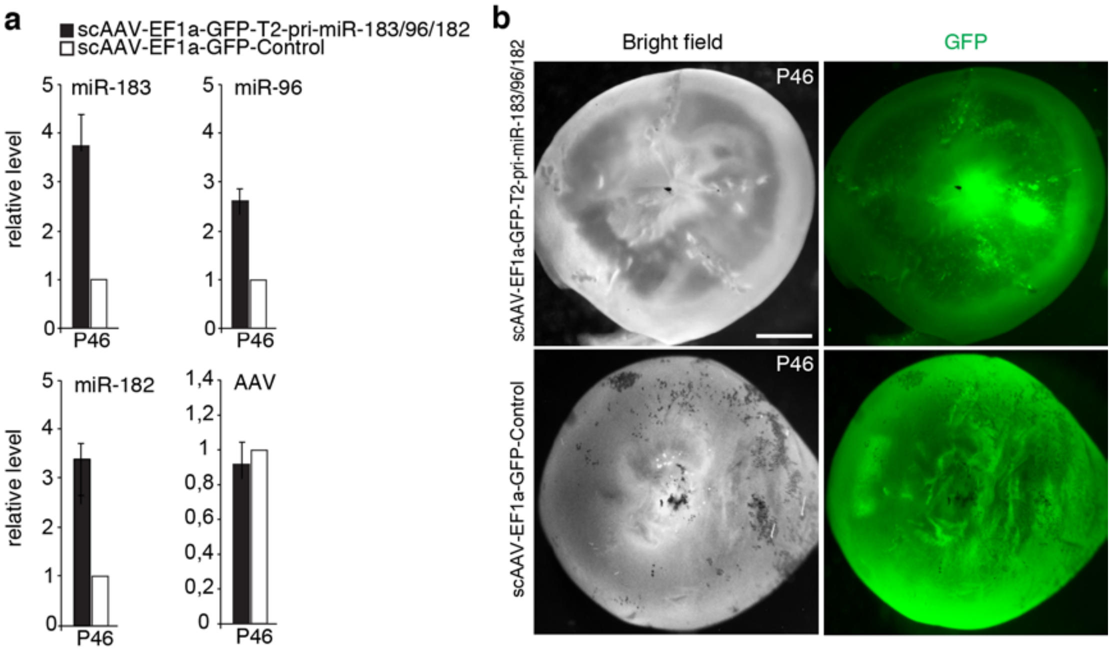

Supplementary Figure 6 | AAV-mediated expression of miR-183/96/182 in adult retina has no effect on the photoreceptor layer architecture.

(**a**) RT-qPCR analysis of miR-183/96/182 and AAV levels in P46 retinas infected with AAVs expressing either T2-pri-miR-183/96/182 or control RNA. Normalized values are means ± s.e.m. *n* = 3. (**b**) Bright field and fluorescent images of P46 retinas expressing T2-pri-miR-183/96/182 or control RNA.

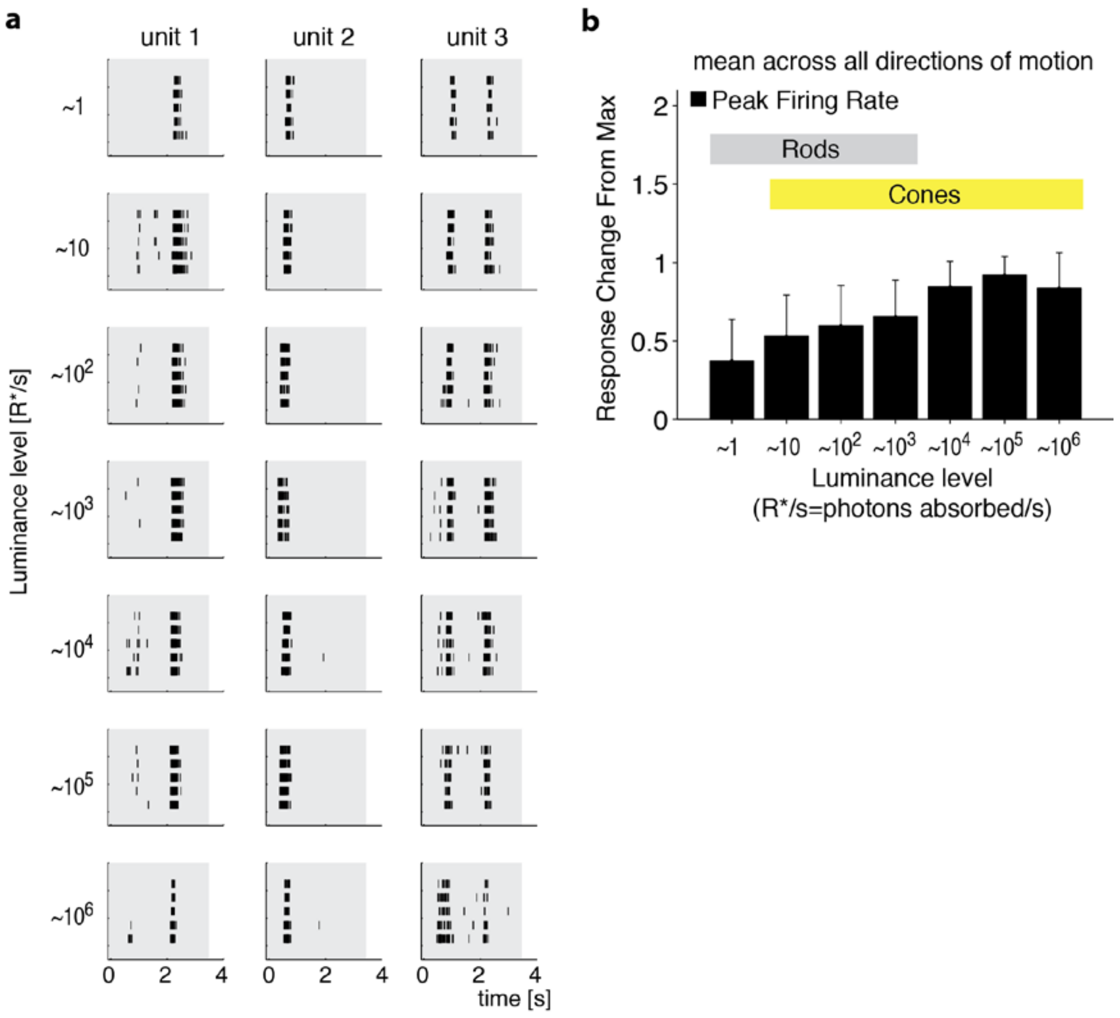

Supplementary Figure 7 | Premature miR-183/96/182 accumulation does not affect photoreceptor function.

(**a**) Raster plots showing responses to a moving bar from three retinal ganglion cells (each column corresponds to a single spiking unit). Unit 1: OFF cell, unit 2: ON cell, unit 3: ON-OFF cell. Photoreceptors were stimulated across seven different luminance levels. Each stimulus was repeated five times. (**b**) Quantification of responses to light stimulation across seven different luminance levels for 59 recorded retinal ganglion cells. The response of each cell across seven different luminance levels was normalized according to the maximum response across all seven luminance levels. Average peak firing rate was computed from spike trains and used to quantify responses. Bars at the top of the plot indicate luminance levels at which rods and cones are active.

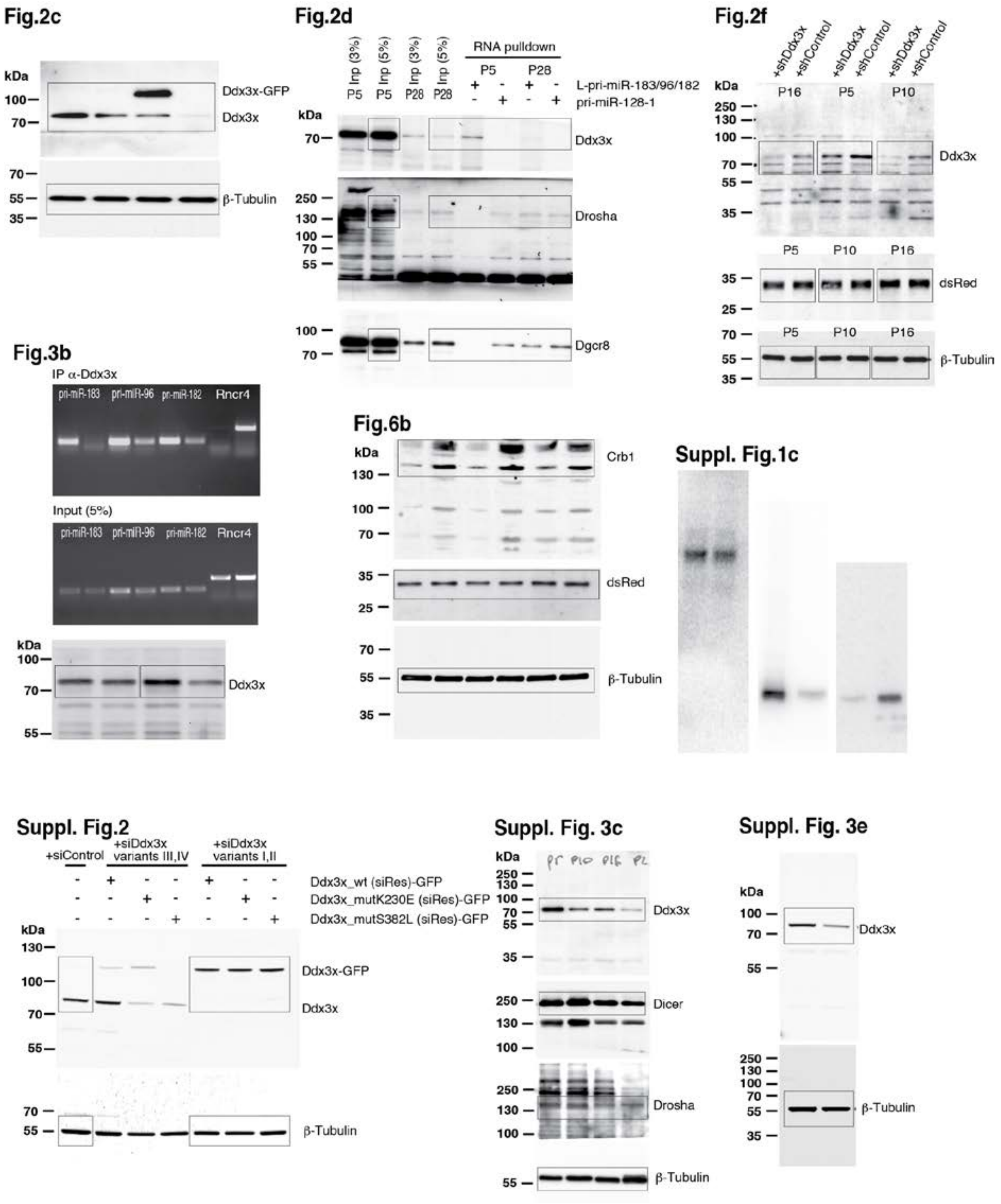

Supplementary Figure 8 | Full-scans of blots and gels with molecular weight (kDa) markers indicated. Corresponding figures and fragments of blots and gels (rectangles) used in them are indicated.

Additional explanatory notes: (**Fig. 6b**) Crb1 is a highly glycosylated protein expressed in several isoforms (153-, 142-, 93-, and 50-kDa) recognized by the used antibody. Expression of all isoforms appears to be regulated by miR-183/96/182. (**Supplementary Fig. 2)** Lanes not incorporated in the figure represent tests with siRNAs (variants III and IV, targeting regions

overlapping with those targeted by variants I and II), which were found to inhibit both endogenous and overexpressed siRes Ddx3x, and therefore were not used in further experiments.

Supplementary Table 1 | Examples of miR genes and neighboring genes encoding lncRNAs in mouse

miR	strand	lncRNA	strand
miR-100/let7a-2/miR-125b-1	+	AK132154	-
miR-1a-1/miR-133a-2	+	B230312C02Rik	-
miR-133a-1/miR-1b/miR-1a-2	+	4930563E18Rik	+
miR-3093/miR-9-1	+	AK141565	+
miR-9-2	+	AK036048;<br>C130071C03Rik	-<br>+
miR-363/miR-92-2/miR-19b-2/miR-20/miR-18b/miR-106a	-	Kis2	-
miR-23b/miR-27b/miR-3074-1/miR-24-1	+	Gm16907	-
miR-128-2	-	2900079G21Rik	+
miR-129b	+	AK140370	-
miR-143/miR-145 cluster	-	LOC545261	+
miR-466..../miR-669a-5 (27 miRNAs)	+	Gm13261	-
miR-301	+	Gm11491	-
miR-483	-	Igf2OS	+
miR-490	+	9330158H04Rik	+
miR-599/miR-875	+	BC048602	-
miR-877	-	Gm8801	+
